# Methanogenic archaea in subsurface coal seams are biogeographically distinct: an analysis of metagenomically‐derived *mcrA* sequences

**DOI:** 10.1111/1462-2920.16014

**Published:** 2022-05-10

**Authors:** Bronwyn C. Campbell, Paul Greenfield, Se Gong, Elliott P. Barnhart, David J. Midgley, Ian T. Paulsen, Simon C. George

**Affiliations:** ^1^ Energy Business Unit Commonwealth Scientific and Industrial Research Organisation (CSIRO) Lindfield NSW 2070 Australia; ^2^ School of Natural Sciences Macquarie University North Ryde NSW 2109 Australia; ^3^ U.S. Geological Survey Wyoming‐Montana Water Science Center Helena MT 59601 USA

## Abstract

The production of methane as an end‐product of organic matter degradation in the absence of other terminal electron acceptors is common, and has often been studied in environments such as animal guts, soils and wetlands due to its potency as a greenhouse gas. To date, however, the study of the biogeographic distribution of methanogens across coal seam environments has been minimal. Here, we show that coal seams are host to a diverse range of methanogens, which are distinctive to each geological basin. Based on comparisons to close relatives from other methanogenic environments, the dominant methanogenic pathway in these basins is hydrogenotrophic, with acetoclastic being a second major pathway in the Surat Basin. Finally, *mcrA* and 16S rRNA gene primer biases were predominantly seen to affect the detection of Methanocellales, Methanomicrobiales and Methanosarcinales taxa in this study. Subsurface coal methanogenic community distributions and pathways presented here provide insights into important metabolites and bacterial partners for *in situ* coal biodegradation.

## Introduction

There are increasing global efforts to reduce greenhouse gas emissions produced by coal fuels in response to climate change. Although renewable energy is generally considered to be the end goal of these efforts, non‐renewable, lower emission alternatives to coal provide useful transition fuels in this process. Coal seam gas is one such transition fuel, because it can provide stable, dispatchable electricity generation using existing infrastructure while producing less greenhouse gas emissions than burning coal (Hardisty *et al*., 2012; Schandl *et al*., 2019). Additionally, coal seam gas does not produce other atmospheric pollutants associated with coal such as particulates and nitrogen or sulfur oxides (Markandya and Wilkinson, 2007).

In recent decades it has become clear that a significant proportion of coal seam gas is actively produced by communities of microbes living within coal seams (Faiz and Hendry, 2006; Flores *et al*., 2008; Strąpoć *et al*., 2011; Golding *et al*., 2013a; Ritter *et al*., 2015). Coal seam gas, which is predominately methane, can be produced either thermogenically via geological processes or biogenically by microbial communities of bacteria and archaea. Although the proportion of biogenic gas varies across deposits, the global biological contribution is estimated to be up to one‐third of the total coal seam gas reserve, with present‐day ongoing biogenic production in some cases (Strąpoć *et al*., 2011; Ritter *et al*., 2015). Previous studies have sought to further enhance present‐day biogenic methane production *in situ* via methods such as adding nutrients to native microbial communities (e.g. see Jones *et al*., 2010; Davis *et al*., 2018, 2019), introducing catalytic compounds (Beckmann *et al*., 2016) or through bioaugmentation: the addition of non‐indigenous microbes to coal seam environments (e.g. see Wang *et al*., 2016). Despite this previous work, many of the processes involved in the biodegradation of coal to methane *in situ*, and the microbes responsible, are poorly understood.

To better understand biogenic production of methane from coal, microbial surveys from water produced at coal seam gas wells have used metagenomic and ribosomal RNA (rRNA) gene sequencing of the 16S subunit to identify many taxa present in coal seams (Strąpoć *et al*., 2011; Vick *et al*., 2018). Although most of these taxa are bacteria with ambiguous functional roles in the communities, the activities of many of the archaea that are present are better understood. Methanogens (methane‐producing archaea) are responsible for the final step of methane production in coal seams. Owing to the presence of methanogens in better‐studied environments, such as the animal gut, anoxic sediments and wastewater, more studies have sought to understand their biology. Methanogens produce methane using the methyl‐coenzyme M reductase (MCR) enzyme, the presence of which can be genetically identified by the amplification of the MCR α subunit (*mcrA*) using polymerase chain reaction (Juottonen *et al*., 2006; Evans *et al*., 2019).

The identification of trends in methanogen distribution within different coal seam‐bearing basins may provide useful inferences for other processes occurring in these environments, however, thus far no studies have examined *mcrA* diversity within coal seams across multiple continents. The dominant methanogens within a coal seam are likely responding to a range of factors, such as the availability of methanogenic precursors (for instance, hydrogen, carbon dioxide, acetate, or other suitable methyl‐containing compounds), available syntrophic partners (e.g. various Deltaproteobacteria, Clostridia, or other taxa) and abiotic factors such as methane partial pressure and groundwater physicochemistry.

The aims of the present study were thus to identify *mcrA* genes within existing, publicly available metagenomic datasets using a tool called ‘Kelpie’ (Greenfield *et al*., 2019), and then to (i) explore the biogeographic distribution of methanogens using the presence of *mcrA* genes, (ii) infer dominant methanogenic pathways in each basin based on close relatives of the *mcrA*‐containing taxa, (iii) determine whether 16S rRNA gene amplicons from the same metagenomes capture this methanogen diversity, and (iv) compare results against some of the previously identified primer pairs used for *mcrA* amplification.

## Results

### mcrA primer selection

Four published *mcrA* primer sets were found from a literature search: ME (Hales *et al*., 1996), ML (Luton *et al*., 2002), MCR (Springer *et al*., 1995) and Angel (mlas‐mod – F and *mcrA*‐rev – R; Angel *et al*., 2012). These primers were adjusted slightly to more closely match the *mcrA* sequences found in the metagenomic datasets (Table 1). Preliminary tests on three of the Australian datasets showed that the MCR and Angel primers were the most effective, with the ML and ME primers failing to recover some *mcrA* sequences found by MCR and Angel, and not finding any additional *mcrA* genes (Supplementary Data Table S1). As a result, the adjusted MCR primer set (Springer *et al*., 1995) and the adjusted Angel primer set (mlas‐mod – F and *mcrA*‐rev – R; Angel *et al*., 2012) were chosen for the extraction of *mcrA* genes from all 13 coal seam metagenomes. In addition to these published primers, equivalent‐region *mcrA* primers based on the two *Bathyarchaeota* sp. reported by the authors of the Surat 6 metagenome (Evans *et al*., 2015) were also trialled on each of the metagenomes in the present study (Supplementary Data Table S2). These primers did not identify any additional taxa across the 13 metagenomes, apart from the two expected *Bathyarchaeota* sp. in the Surat 6 metagenome. Hereafter all analyses refer to data obtained from the MCR and Angel primer sets.

**Table 1:** *mcrA* primer sets adjusted for improved use with Kelpie.

Primer set	Adjusted sequence (5′ to 3′)	Original reference
MCR	Forward‐TWYGAYCARRTHTGGYT	Springer *et al*. (1995)
	Reverse‐ACRTTCATNGCRTARTT	
ME	Forward‐GCMATGCARATNGGWATGWS	Hales *et al*. (1996)
	Reverse‐TCATKGCRTAGTTDGGRTAGT	
ML	Forward‐GGYGGWGTMGGNTTCACMCARTAYGCWACNGC	Luton *et al*. (2002)
	Reverse‐TTCATTGCRTAGTTWGGRTAGTT	
mlas‐mod – F *mcrA*‐rev – R^a^	Forward‐GGYGGYGTMGGDTTCACMCARTA	Angel *et al*. (2012)
	Reverse‐CRTTCATNGCRTARTTNGGRTAGT	

Adjusted bases are underlined.

^a^ Referred to as the ‘Angel’ primer set in the present study, after the lead author of the 2012 study.

### mcrA diversity

**Fig. 1:**
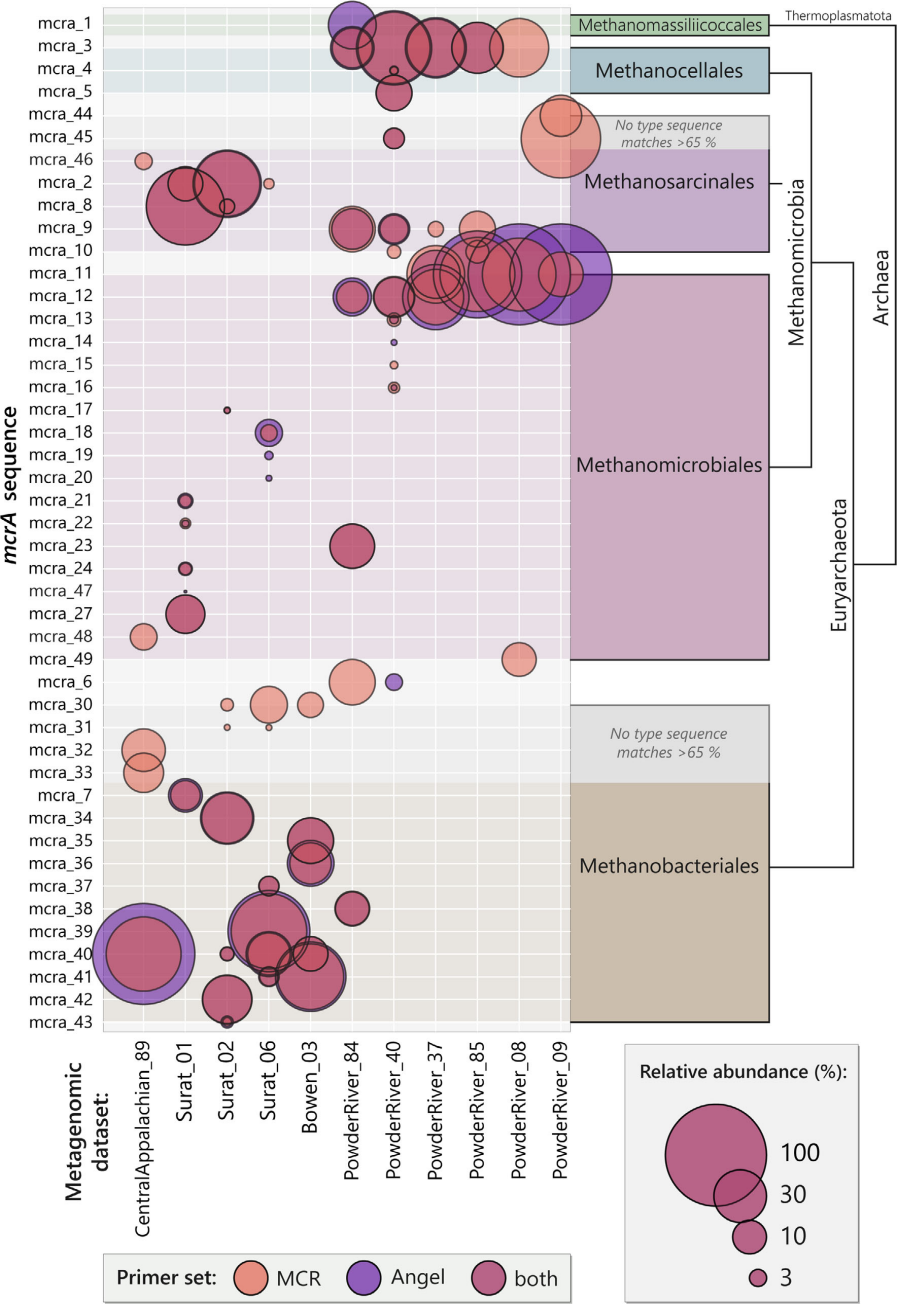
Proportion of distinct *mcrA* sequences within each metagenomic dataset as detected with the MCR primer set and Angel primer set. Phylogenetic groupings of *mcrA* sequences by BLAST type sequence matching are provided down to the class level.

**Fig. 2:**
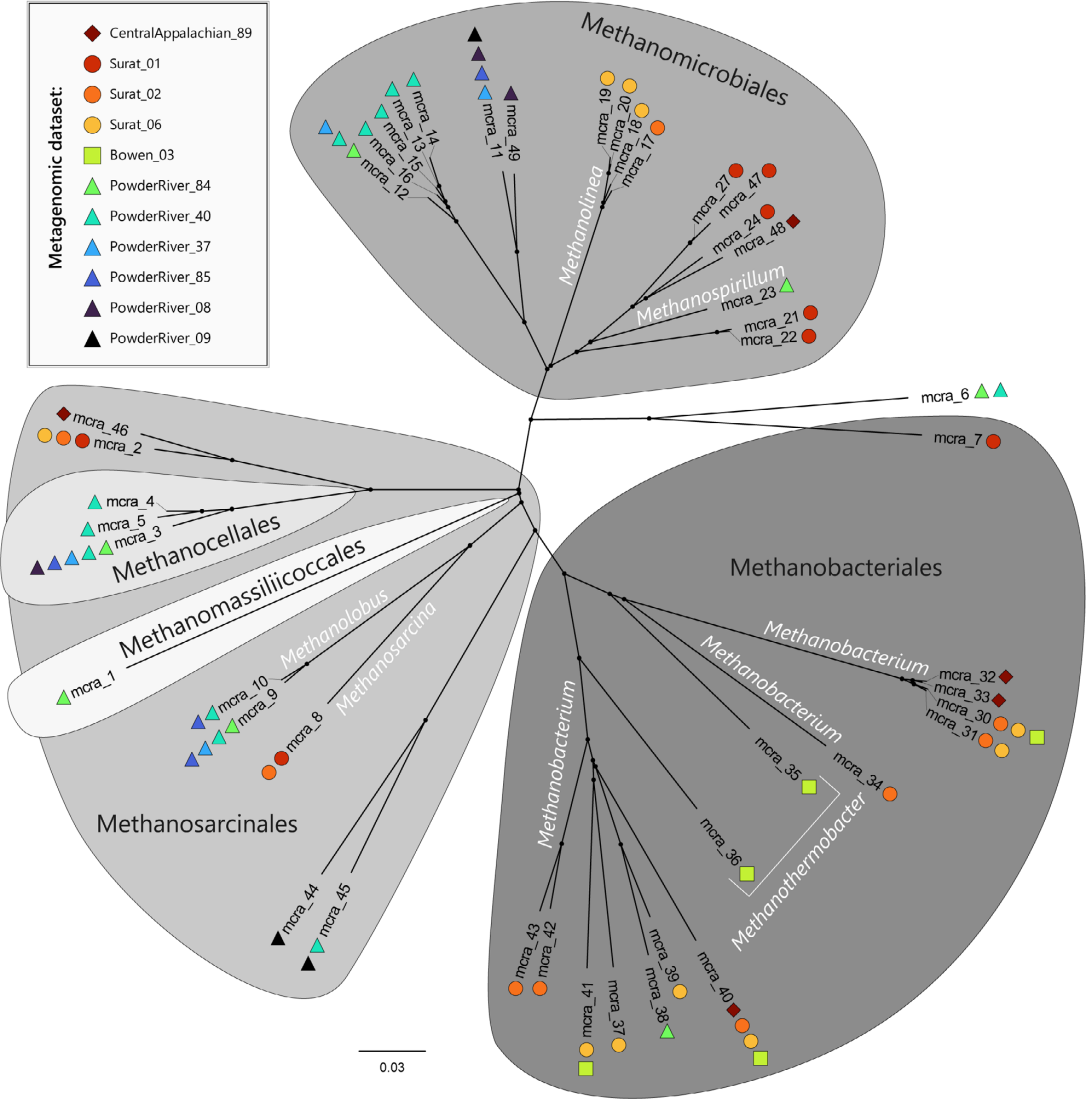
Phylogenetic tree of the *mcrA* sequences merged from those produced by Kelpie using both the Angel and MCR primer sets. The 0.03 scale bar indicates the number of nucleotide changes per site.

Prior to merging operational taxonomic units (OTUs) across the 13 metagenomic datasets, 56 distinct *mcrA* sequences were retrieved using the Angel primer set, while 86 distinct *mcrA* sequences were obtained using the MCR primer set. Merging sequences from all metagenomic datasets and primers produced a total of 45 distinct *mcrA* sequences (Figs 1 and 2; Supplementary Data Table S3). Most of the *mcrA* sequences were found with both the Angel and MCR primers, but some were only found using one of the primer sets (Supplementary Data Table S4). For example, mcra_1 was detected only by the Angel primer set, whilst mcra_30 was only detected using the MCR primer set (Supplementary Data Table S3). Some *mcrA* gene types were only distantly related to all other types (Fig. 2), for example, mcra_1. In contrast, other *mcrA* variants were members of closely related groups. For instance, *mcrA* sequences 12 through 16, all from the Powder River Basin, were all very closely related to each other, and are related to other Methanomicrobiales previously observed in fens or lake sediment (Fig. 2; Supplementary Data Table S3).

**Fig. 3:**
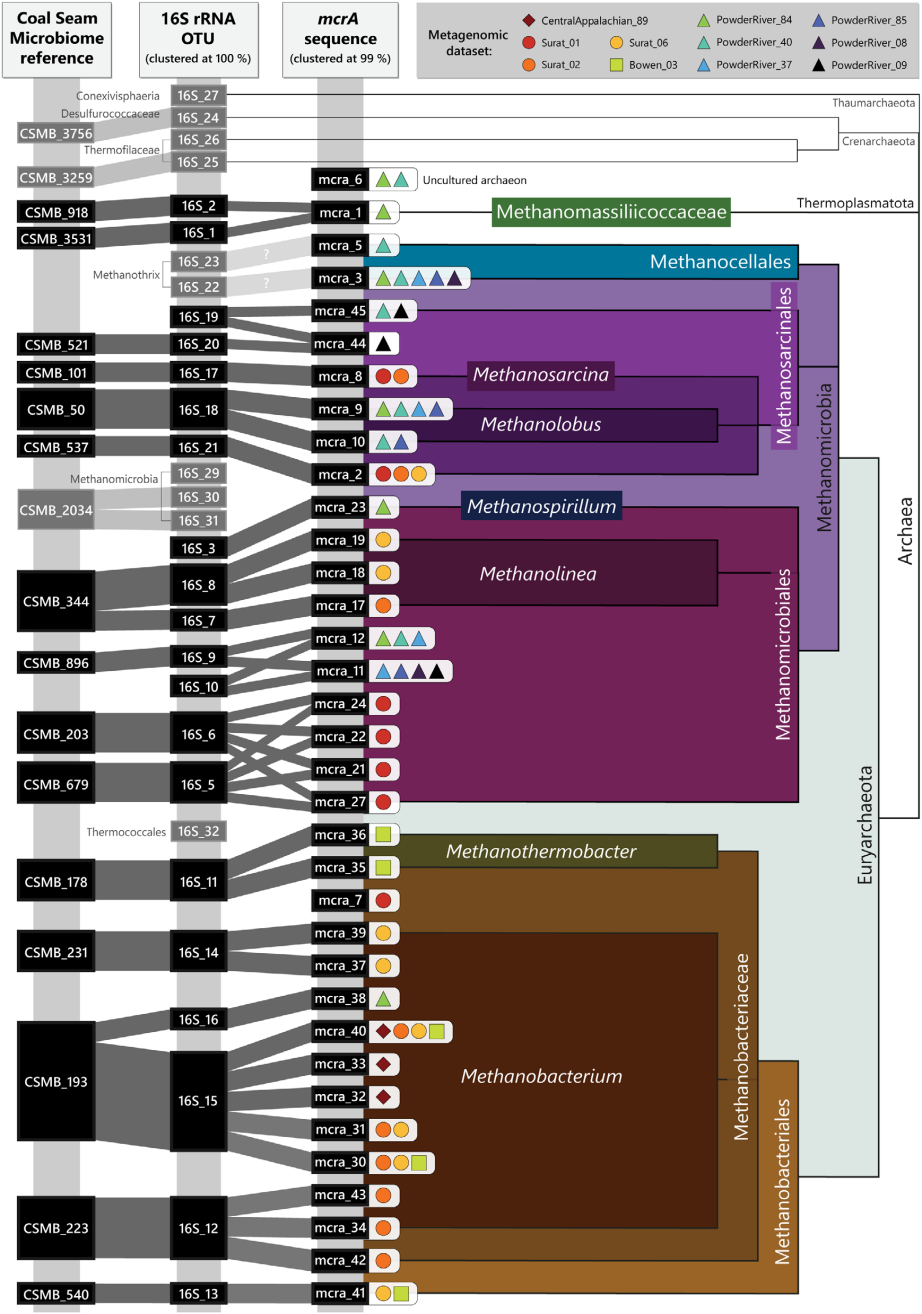
Most plausible *mcrA* sequence and 16S OTU matches based on correlating the presence of both sequences in each dataset and corresponding NCBI BLAST type sequence matches (Fig. 2; Supplementary Data Table S3; Supplementary Data Table S10; Supplementary Data Fig. S2). Coal Seam Microbiome (CSMB) reference set matches mapped directly to the 16S rRNA gene OTUs shown as linked, using a >97% ident. All *mcrA* sequences with total reads of five or less were excluded from this figure.

*mcrA* genes were detected in all samples except Powder River 10 and Powder River 50 (Fig. 1; Supplementary Data Table S5). For those *mcrA* genes detected, richness ranged from 3–11 taxa, with Powder River 8 and Powder River 9 having the fewest *mcrA* types, and Powder River 40 having the most. From a phylogenetic perspective, *mcrA* diversity in the present study is distributed across two phyla (Euryarchaeota and Thermoplasmatota), three classes and five orders (Figs 1 and 3). In terms of individual datasets, Powder River 84 had the highest biodiversity (Simpsons 1‐D = 0.83), while Powder River 9 had the lowest biodiversity (Simpsons 1‐D = 0.42; Supplementary Data Table S6).

### Archaeal 16S rRNA gene diversity

Using the Earth Microbiome Project 16S rRNA gene primers (Apprill *et al*., 2015; Parada *et al*., 2016) to guide Kelpie retrieved 11 320 archaeal sequences clustered into 34 zero‐radius OTUs. Regions encoding for 16S rRNA were detected in all samples in the present study, although no archaeal sequences were detected in Powder River 10. Archaeal taxa from four different phyla (Thaumarchaeota, Crenarchaeota, Thermoplasmatota and Euryarchaeota) were detected, and these were further distributed into six classes and eight orders (Supplementary Data Table S7; Supplementary Data Fig. S1). Taxa from Thaumarchaeota (16S_27) and Crenarchaeota (16S_24, 16S_25 and 16S_26) were assembled from the Surat 6 metagenomic dataset only and did not correlate to any *mcrA* genes. The *Thaumarchaeota* sp. (16S_27) is closely related to sulfur and iron‐reducing taxa (Kato *et al*., 2020). Similarly, the *Crenarchaeota* spp. is closely related to taxa capable of processes such as sulfur respiration (16S_24, 16S_25 and 16S_26; Zillig *et al*., 1982, 1983) or carbon dioxide and hydrogen production (16S_25 and 16S_26; Kochetkova *et al*., 2020), rather than methanogenesis.

### mcrA community comparisons

**Fig. 4:**
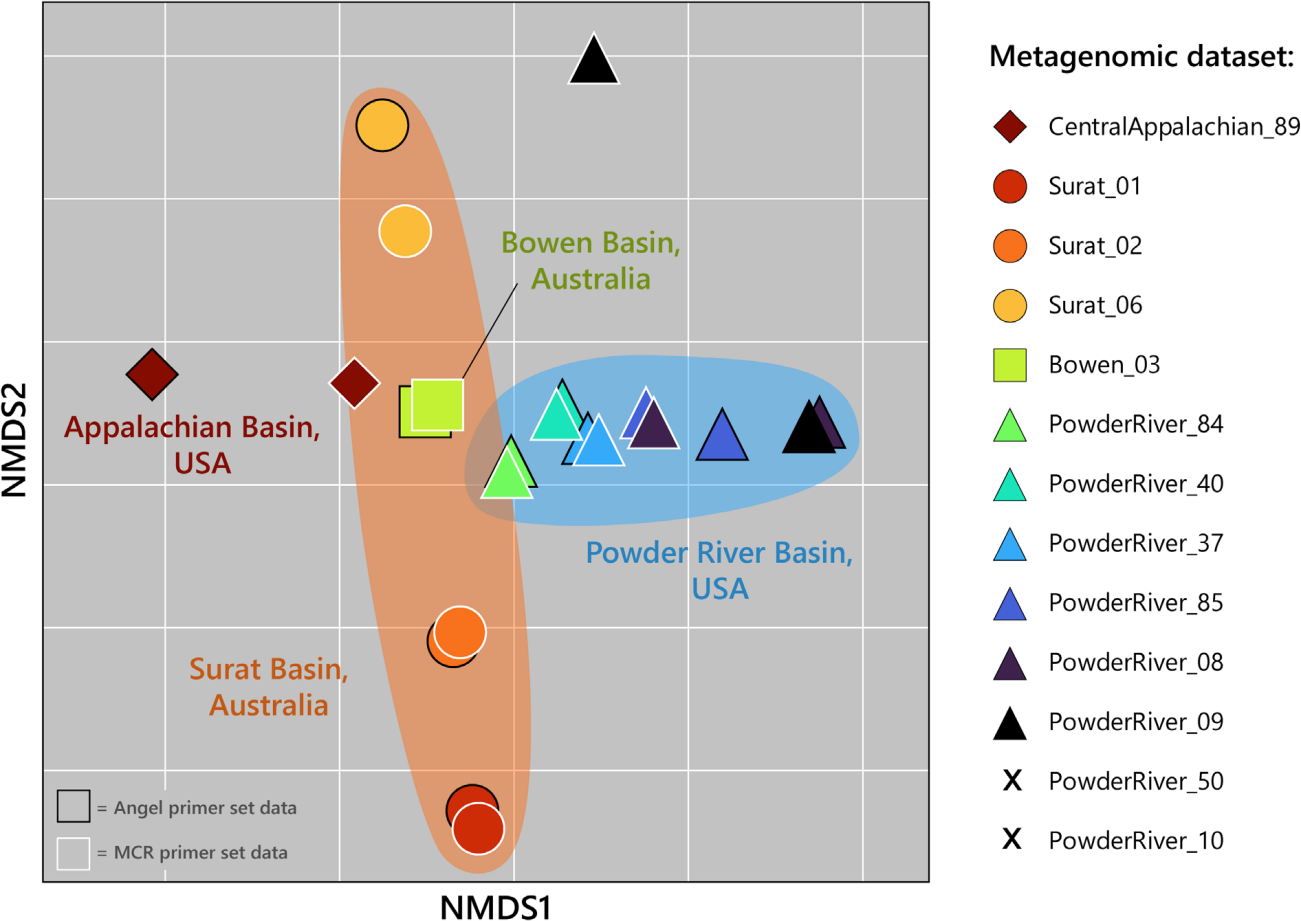
Two‐dimensional NMDS plot of the *mcrA* sequences detected in the metagenomic datasets selected for this study. No *mcrA* sequences were detected in the Powder River 50 and Powder River 10 datasets.

Non‐metric multidimensional scaling (NMDS) ordination of the archaeal *mcrA* gene distribution by site demonstrated a clear separation of the Central Appalachian 89 dataset from other sites (Fig. 4). In contrast, *mcrA* communities from the Powder River Basin samples were variable, with some distinct samples (e.g. Powder River 8 and 9) and others which appear to have somewhat more variable *mcrA* communities. These differences between communities by sampling sites were supported by phylogenetic differences between the *mcrA* sequences observed in the present study (Fig. 1). For example, the Powder River Basin appears to be dominated by *mcrA* genes from Methanocellales, Methanomicrobiales and Methanosarcinales, whereas the Australian Basins and central Appalachian Basin appear to be dominated by Methanobacteriales and Methanosarcinales (Fig. 1; Supplementary Data Table S3).

Although a greater number of distinct *mcrA*‐containing taxa were detected with the *mcrA* primers, the 16S rRNA gene sequences covered a wider range of phylogenetic groupings of archaeal taxa (Figs 1 and Fig. 3; Supplementary Data Fig. S1). For example, the 16S rRNA gene primers detected sequences from the Thaumarchaeota and Crenarchaeota phyla, neither of which were detected by the *mcrA* primer sets. Overall, most *mcrA* genes were matched to one or more 16S rRNA gene OTUs, and vice versa (Fig. 3). From both the *mcrA* and 16S OTUs, the dominant classes were Methanomicrobia and Methanobacteria.

### Water chemistry description

In broad terms, water chemistry for the samples examined here (Surat 1, Surat 2 and Bowen 3) had similar characteristics (Supplementary Data Table S8). All samples were alkaline (pH 8.4–8.8), moderately brackish (electrical conductivity of 1900 to ~9400 μS cm^−1^), low in nitrogen (<2.2 ppm) and very low in both phosphorus (≤0.04 ppm) and sulfate (<25 ppm). Water chemistry for the Powder River Basin metagenomes has also been provided in Supplementary Data Table S9 for comparison (Barnhart *et al*., 2016).

## Discussion

This study presents the first analysis of *mcrA* gene distribution across multiple coal‐bearing basins in Australia and the United States of America. Data presented here suggest both the Angel and MCR primers provide good representation of *mcrA* gene diversity and abundance in coal seam environments. For ease of discussion, the authors hereafter consider *mcrA* diversity to be a proxy for methanogen diversity, though it should be noted that the two are not completely interchangeable. Overall, the results demonstrate that different coal‐bearing basins support distinct and mostly unique communities of methanogens, with relatively few shared taxa occurring across basins. The distinctiveness of *mcrA* gene diversity by basin is also supported by the archaeal 16S rRNA gene region diversity, though none of the primer sets captured the full range of diversity, which was best achieved by using both the *mcrA* and 16S rRNA gene results in combination.

### mcrA diversity in coal seam environments

Diversity of *mcrA* genes across coal seams is generally greater than for other methanogenic environments such as animal guts and sediments, suggesting a relatively higher diversity of degradation strategies may be occurring within coal seam environments. Within the guts of buffalo, cattle and sheep, for example, *mcrA* genes have diversity indices that are generally much lower than those in the present study (Lwin *et al*., 2012; Snelling *et al*., 2014). This is consistent with low bacterial diversity in animal guts relative to coal seams (Ley *et al*., 2008; Vick *et al*., 2018). Similarly, *mcrA* diversity within subglacial and gas hydrate sediments is also lower relative to the taxa detected in the present study (Marchesi *et al*., 2001; Boyd *et al*., 2010), while diversity levels more similar to the present study have been found within hydrothermal sediments (Dhillon *et al*., 2005). There are a few possible explanations for this higher diversity.

Higher diversity in hydrothermal sediments and coal seams may be a consequence of lower nutrient availability (Wheat *et al*., 1996; Davis *et al*., 2018), as observed in soil microbial communities (Zeng *et al*., 2016; Wang *et al*., 2018). In the case of coal seams, higher diversity may also result from their relative isolation from external influences, due to formation water residence times varying from tens of thousands of years in the Powder River Basin (Bates *et al*., 2011) to millions of years in the Surat Basin (Siade *et al*., 2018). A further possibility is that the high diversity of the *mcrA* genes may be a function of ecological niches of the coal‐degrading bacteria capable of forming syntrophic partnerships with methanogens, or may be a long term consequence of distinct founder effects across separate coal seam environments during influx of meteoric water after initial coalification (Waters *et al*., 2013; Golding *et al*., 2013a). Nevertheless, further investigation of coal seam degradation processes would benefit from consideration of the wide range of observed methanogen diversity in order to gain a more comprehensive understanding of coal to methane transformations.

### Distinctiveness by geological basin

Unsurprisingly, the *mcrA* genes discovered are more similar between basins that have possible interconnectivity. For instance, Surat Basin *mcrA* profiles are most similar to the Bowen Basin, whilst the central Appalachian Basin and Powder River Basin are markedly distinct from each other (Figs 1 and Fig. 4; Supplementary Data Figs S1 and S2), and both are distinct from the Australian basins. Indeed, the central Appalachian Basin and Powder River Basin share no common taxa, despite being situated on the same continent. They are, however, approximately 2000 km distant from one another, with no potential for interconnectivity between aquifers or overlapping groundwater recharge locations. In contrast, similarities between taxa identified in the Australian basins may result from shared groundwater factors arising from their geographic proximity.

The Surat Basin overlies the Bowen Basin, and this relationship combined with their similar methanogenic taxa suggests either some interconnectivity of groundwater systems or shared groundwater recharge regions. The Australian metagenomic datasets used in the present study were originally sampled from the Walloon Subgroup (also known as the ‘Walloon Coal Measures’) in the Surat Basin, and the Bandanna Formation in the Bowen Basin (Supplementary Data Table S5). Although the Walloon Subgroup overlies the Bandanna Formation, they are separated by numerous other geological formations. Two of these, the Evergreen Formation and the Rewan Group, are considered to be substantially thick (averaging up to 300 m) and laterally persistent aquitards, and thus likely allow little to no interconnectivity between the Walloon Subgroup and the Bandanna Formation (Queensland Water Commission, 2012). Consequently, the more likely cause of the similar diversity of methanogenic taxa in these formations is a shared groundwater recharge location, and indeed the Bandanna Formation is likely primarily recharged where it outcrops along the northern boundary of the Surat Basin, along the Great Dividing Range (Queensland Water Commission, 2012). Environments more proximal to the surface, such as soils, alluvial sediments and other shallow aquifer regions, may therefore host a microbial seed bank from which microbes are dispersed into the subsurface aquifers of this region (Gittins *et al*., 2021; Mestre and Höfer, 2021). Widespread metagenomic sampling of these basins, detailed metadata regarding sample locations and depth, and a more thorough hydrogeological analysis of the interconnectivity and recharge of these basins would be valuable in confirming the existence of a recharge‐related microbial seed bank in this region.

### Common methanogens of the present study and co‐occurrences within the Coal Seam Microbiome (CSMB) reference set

The methanogen most commonly detected in subsurface coal seams is the uncultured *Methanobacterium* sp. CSMB_193 (mcra_40; 16S_15, 16S_16 this study). Analyses presented here indicate that *Methanobacterium* sp. mcra_40 likely corresponds to the 16S_15 OTU, which is part of CSMB_193 and further matches (>97%) to the 16S_16 OTU (Fig. 3; Supplementary Data Table S10; Supplementary Data Fig. S1). Not only was *Methanobacterium* sp. CSMB_193 the most abundant *mcrA* taxon in the central Appalachian Basin dataset, but it was detected in all four basins of the present study (Figs 1 and 3; Supplementary Data Fig. S1). The uncultured *Methanobacterium* sp. CSMB_193 are the most commonly found coal seam methanogens globally, being present in the Sydney, Surat and Bowen basins in Australia, the Jingmen‐Dangyang Basin in China, the Ishikari Basin in Japan, and the Cherokee, Powder River and central Appalachian basins in the USA (Supplementary Data Table S11; Vick *et al*., 2018).

The other most notable *mcrA* genes in the present study were *Methanocellales* sp. mcra_3 (no 16S rRNA gene match), *Methanolobus* sp. mcra_9 (16S_18; CSMB_50) and *Methanomicrobiales* sp. mcra_11 (16S_9/16S_10; CSMB_896) respectively (Fig. 3; Supplementary Data Table S10; Supplementary Data Table S11). These methanogens were the most common across those Powder River Basin datasets which contained detectable *mcrA* genes. The *Methanomicrobiales* sp. mcra_11, if it does indeed correspond to taxa within CSMB_896, has previously been detected in the Surat, Bowen and Sydney basins in Australia, and the Jingmen‐Dangyang Basin in China (Vick *et al*., 2018). The *Methanolobus* sp. mcra_9, if corresponding to CSMB_50, appears to be even more widespread and has thus far been detected in the Surat, Bowen, and Sydney basins in Australia, the Jingmen‐Dangyang and Ordos basins in China, the Ishikari Basin in Japan, and the Cherokee and Powder River basins in the USA (Vick *et al*., 2018).

From the remaining methanogens able to be linked to the coal seam microbiome (CSMB) set, several have been reported to be enriched in communities grown on specific coal components or organic compounds (Vick *et al*., 2019; Campbell *et al*., 2021). From a study of the degradation of specific organic fractions in coal, *Methanocalculus pumilus* CSMB_203 (Central Appalachian 89, Surat 1) was found on benzoate and solvent‐extracted coal, *Methanosaeta* sp. CSMB_537 (Central Appalachian 89, Surat 1 and 2) was found on unaltered coal, and *Methanospirillum hungatei* CSMB_679 (Surat 1) was found on the polar compounds extracted from coal (Vick *et al*., 2019). Another study on the degradation of aromatic compounds associated with coal again found the *Methanosaeta* sp. CSMB_537 enriched on phenol, benzoate and syringate, and *Methanomassiliicoccus* sp. CSMB_3531; Powder River 37, 50 and 84) enriched on ethylbenzene (Campbell *et al*., 2021). Potential organic matter variations which lead to the production of substrates favourable to coal seam methanogens can be inferred using studies such as the above. For example, *Methanosaeta* sp. CSMB_537 is present in the Surat 1 and Surat 2 datasets of the present study, thus aromatic compounds with hydroxyl and carboxyl functional groups from coals in the Surat Basin may be important organic compounds for the Surat Basin methanogenic microbial communities.

### Putative methanogenic pathways

The *mcrA* types detected in the present study were primarily associated with carbon dioxide‐reducing methanogens in the Methanocellales, Methanomicrobiales and Methanobacteriales (Kallistova *et al*., 2017; Burdukiewicz *et al*., 2018; Evans *et al*., 2019). This result suggests that carbon dioxide, hydrogen and/or formate are likely to be important end‐products from the degradation of the coal by the bacterial communities in the coal seams, before being utilized by the methanogenic communities.

In addition to the large carbon dioxide‐reducing taxa, several *Methanosarcinales* spp. were identified, including a *Methanosaeta* sp. mcra_2 (16S_21; CSMB_537) and *Methanosarcina* sp. mcra_8 (16S_17; CSMB_101) from the Surat Basin datasets. *Methanosaeta* species are strictly acetoclastic methanogens, while *Methanosarcina* species are metabolically flexible and can use hydrogenotrophic, acetoclastic or methylotrophic pathways for methanogenesis (Kallistova *et al*., 2017). The overall highest diversity and relative abundance of *Methanosarcinales* spp. were best represented by 16S rRNA genes across the Powder River Basin datasets, where *Methanolobus psychrophilus* (16S_18; CSMB_50) and *Methanothrix soehngenii* (16S_22; no CSMB match) were the dominant Methanosarcinales taxa. *Methanolobus* spp. have been previously identified in the Powder River Basin (Barnhart *et al*., 2013), and are commonly recorded utilizing mono‐, di‐ or trimethylamines, dimethyl sulfide, or alcohols for methanogenesis, and are unable to use acetate (Burdukiewicz *et al*., 2018). In contrast, *Methanothrix* spp. are prominent acetoclastic methanogens, growing most effectively when acetate concentrations are very low (<1 mM) (Kallistova *et al*., 2017).

Despite the prominence of these *Methanothrix* type sequence matches in the 16S rRNA gene results, there are no corresponding *mcrA* type sequence matches (Fig. 3). Similarly, the Methanocellales type sequence matches are abundant in the *mcrA* results yet have no corresponding 16S rRNA gene matches. Given both the distribution patterns of these *Methanothrix* and Methanocellales sequences across the 13 metagenomes, and the consideration of the non‐type sequence matches from the BLAST nt database, these two distinct sequence types appear to be from the same methanogenic group, likely *Methanothrix*. This inconsistency causes uncertainty when attempting to determine likely methanogenic pathways of close relatives, and is a disadvantage of attempting to link different sequencing regions via taxonomic naming conventions. Axenic cultures or genome‐resolved metagenomic analysis would be beneficial for clarifying that these *mcrA* and 16S rRNA gene sequences are indeed from the same methanogen, as well as clarifying the methanogenic pathways available to it and the appropriate taxonomic classification.

### Comparison to stable isotope inferences

Predicted methanogenic pathways using methanogenic taxa identified here align poorly to stable carbon and hydrogen isotopes signature predictions reported for these basins, which is in agreement with previous reports that the broad application of stable isotope ratios for methanogenic pathway predictions is unreliable in the coal seam environment (Bates *et al*., 2011; Vinson *et al*., 2017). Stable isotope ratios of carbon and hydrogen in methane (δ^13^C_CH4_ vs. δD_CH4_), among other widely used ratios are regularly used to infer whether acetoclastic/methylotrophic, carbon dioxide‐reduction or mixed microbial methanogenic pathways are being utilized by methanogens in a given environment (Whiticar *et al*., 1986; Whiticar, 1999). Methane isotope data from the Surat Basin places it between thermogenic and carbon dioxide reduction for methanogenesis (Golding *et al*., 2013a; Golding *et al*., 2013b; Baublys *et al*., 2015), whereas taxa from the present study indicate that acetoclastic methanogenesis likely represents at least part of the methanogenic potential in this basin. The Powder River Basin is a little better, as the methane stable isotope inference of the predominantly acetoclastic/methylotrophic pathway here (Flores *et al*., 2008; Strąpoć *et al*., 2011) is broadly in agreement with the presence of methanogens such as *M*. *psychrophilus* (16S_18; CSMB_50) and *Methanomassiliicoccaceae* spp. (mcra_1; 16S_1; 16S_2; CSMB_918; CSMB_3531) in the present study, though their relative abundance is generally low. The majority of the Powder River Basin methanogens are primarily associated with either the carbon dioxide reduction or acetoclastic pathways, with the only probable acetoclastic methanogen (16S_22; 16S_23; Supplementary Data Table S10; Supplementary Data Fig. S1) likely favouring very low concentrations of acetate (Kallistova *et al*., 2017). In the case of the Bowen Basin, methane isotope ratios vary widely but remain within or between the carbon dioxide reduction or thermogenic regions (Golding *et al*., 2013b), which aligns with taxa in the present study.

Stable isotope signature discrepancies relative to observed methanogenic taxa have previously been explained as resulting from these stable isotope pathway predictions being unable to encompass the multitude of alternative pathways and environment‐specific pressures that characterize methanogenic communities (Conrad, 2005; Vinson *et al*., 2017). Non‐methanogenic processes such as sulfate reduction (relevant to the Powder River Basin here), mixing of biogenic and thermogenic methane, and equilibration of methanogenic precursor isotopic ratios with the coexisting formation water are all potential sources of these observed discrepancies in accurate prediction of methanogenic pathways (Vinson *et al*., 2017). Methods for more reliable inferences of methanogenic pathways in coal seams include the use of metagenomics (as in the present study) or *mcrA*‐guided PCR.

### mcrA primers

Results presented here indicate that the MCR (Springer *et al*., 1995) and Angel (mlas‐mod – F and mcra‐rev – R; Angel *et al*., 2012) primer sets are more suited to profiling methanogens from coal seam environments than the ME (Hales *et al*., 1996) and ML (Luton *et al*., 2002) primer sets. Whilst the ME and ML primers did not perform well in the coal seam environment, this may be a result of the types of methanogens present in these environments and they may be valuable in other settings.

Variations in sulfate concentrations and primer bias may explain the very low abundance or absence of *mcrA* genes in the Powder River 10 and Powder River 50 results, as well as the absence of archaeal 16S rRNA encoding regions in the Powder River 10 results. Sulfate concentrations associated with the Powder River 10 dataset sampling location (Nance coal seam; Supplementary Data Table S9) are the highest recorded from the 13 total metagenomic datasets and thus it seems likely that methanogens are indeed very rare to absent, which is further supported by the very low methane concentrations also recorded for this dataset. Powder River 50 has relatively high sulfate concentrations, yet 16S rRNA genes were detected from *Methanomassiliicoccaceae* sp. 16S_1 (CSMB_3531) and *Methanolobus psychrophilus* 16S_18 (CSMB_50) (Supplementary Data Fig. S1). *Methanomassiliicoccaceae* spp. in the Powder River Basin have been previously recorded as capable of tolerating higher than usual sulfate concentrations for methanogenesis (Schweitzer *et al*., 2019; Smith *et al*., 2021), though as a non‐Euryarchaeota methanogen they may be more susceptible to *mcrA* primer bias. The relatively low abundance of *M*. *psychrophilus* 16S_18 in this dataset may have been more readily detected by its 16S rRNA gene due to higher copy numbers relative to the *mcrA* gene copy numbers.

Overall, combined data from the MCR and Angel primers were able to detect a higher diversity of taxa than the 16S rRNA gene V4 primer set (Apprill *et al*., 2015; Parada *et al*., 2016) in most of the metagenomic datasets, especially within the Methanomicrobiales class (consistent with Castro *et al*., 2004), and together the primers were better at identifying more *mcrA* types than alone. Some taxa, however, were better identified using 16S rRNA gene primers (particularly *Methermicoccaceae* spp. and *Methanothrix* spp.). This may be caused by greater copy numbers for the 16S rRNA gene region relative to the *mcrA* gene in these taxa: for example, some other Methanosarcinales taxa contain three 16S rRNA gene copies and only one *mcrA* copy (Nunoura *et al*., 2006). Another explanation for this discrepancy is divergence of the *mcrA* gene. For example, the Surat 6 dataset contains previously identified bathyarchaeotal methanogens which are not detected by the *mcrA* primers generally suited to the Euryarchaeota (Evans *et al*., 2015). Similarly, *mcrA* primer mismatches may be occurring within the Methermicoccaceae and *Methanothrix* taxa. The lack of comprehensive sampling by any single *mcrA* (or 16S rRNA gene) primer set highlights the benefit of using tools such as Kelpie with metagenomic datasets, as this reduces primer bias and provides a more thorough depiction of relative abundance and diversity in these communities.

### Conclusions and future work

By comparing the presence and diversity of inter‐primer *mcrA* and 16S rRNA gene sequences across multiple basins, the present study highlights both the distinctiveness and variability of methanogenic taxa across geographically distinct basins. Taxa from the Methanomicrobia and Methanobacteria classes were dominant in all four basins, with the orders Methanomicrobiales, Methanobacteriales, Methanosarcinales and Methanocellales associated with most of the identified *mcrA* genes. The *mcrA* primer sets were able to detect the largest range of distinct taxa (45 from the *mcrA* sets vs. 34 from the archaeal 16S rRNA gene set, some of which were not methanogenic), however, none of the primers comprehensively sampled all methanogens detected by the others. Future studies would benefit from consistent and sample‐specific location data for comparison with variables such as modelled basin hydrogeology to determine interconnectivity or isolation of different regions. Finally, work to more broadly characterize the genomes, or isolate coal seam methanogens would be valuable in expanding our understanding of these important taxa (such as Evans *et al*., 2015; Mayumi *et al*., 2016; Kurth *et al*., 2021), particularly for the uncertain Methanocellales/*Methanothrix* sequences.

## Experimental procedures

The NCBI GenBank (https://www.ncbi.nlm.nih.gov/genbank/), Joint Genome Institute (https://jgi.doe.gov/), European Bioinformatics Institute (https://www.ebi.ac.uk/), European Nucleotide Archive (https://www.ebi.ac.uk/ena/browser/) and metagenomics RAST (https://www.mg-rast.org/) sequence databases were each searched for Whole Genome Shotgun (WGS) entries fitting within specific criteria to ensure quality and consistency. Each WGS entry selected for use in the present study was required to: (i) have come from a subsurface coal seam (unamended with microbes from other environments), (ii) have a recorded location for a specific geological basin, (iii) be sequenced using Illumina (MiSeq, HiSeq or NovaSeq), and (iv) be available as unassembled sequence reads. Thirteen metagenomic datasets were found to fit the above criteria (Supplementary Data Table S5).

### Kelpie

*mcrA* gene sequences were extracted from the metagenomic datasets using Kelpie (Greenfield *et al*., 2019). Kelpie takes a pair of primer sequences and extracts the corresponding between‐primer regions from a metagenomic dataset, working much like an *in silico* PCR tool. A literature search resulted in the identification of the four *mcrA* primer pairs shown in Supplementary Data Table S12. These primers were trialled with Kelpie to determine which ones were most effective at finding *mcrA* genes, and whether any small modifications were needed to make them more effective. Small changes were made to the published primers to improve their effectiveness by reducing the number of off‐target matches, as shown in Table 1.

All 13 metagenomic datasets were then processed using the chosen Angel and MCR primers to extract the *mcrA* genes present in the samples. Kelpie was also run over the same datasets using the 16S rRNA gene V4 primers 515F and 806R (Apprill *et al*., 2015; Parada *et al*., 2016) to determine community composition.

The individual sets of *mcrA* gene sequences extracted from each metagenomic dataset using each primer pair were clustered at 99% identity with USEARCH (v11) to remove minor variations and sequencing errors. The clustered sequences from all datasets were then merged into two files, one for those extracted using the Angel primer pair, and another for those found with the MCR primer pair. The region of the *mcrA* gene targeted by the Angel primer pair is included within the MCR‐selected region, so both sets of primer‐specific sequences could be merged into a single file. These sequences were then sorted by length and clustered again at 99% to generate a set of consensus sequences across all 13 metagenomic datasets and both primer sets. The script used for this process is provided in the supplementary data.

Once these *mcrA* genes were obtained from the metagenomic datasets, NCBI BLAST (https://blast.ncbi.nlm.nih.gov/Blast.cgi) was used to identify close relatives of the *mcrA* sequences from within the GenBank nucleotide collection. The *mcrA* sequences (and 16S rRNA gene sequences) are provided in FASTA format files in the supplementary data.

### mcrA community comparisons

*mcrA* genes from different datasets were analysed using NMDS in Past v3 (Hammer *et al*., 2001). Co‐ordinate data from Past were exported to the matplotlib module (Hunter, 2007) in Python v.3.5 (https://www.python.org/) to produce plots. The *mcrA* genes were also clustered to produce a neighbour‐joining tree using Clustal Omega (Sievers *et al*., 2011) and FigTree v1.4.4 (http://tree.bio.ed.ac.uk/software/figtree/). NMDS and tree data were annotated using the Inkscape and Adobe Illustrator vector graphics software.

Additionally, the BLAST matches were used to link the *mcrA* sequences with the 16S rRNA gene OTUs in order for comparisons to be made with other studies, including the CSMB reference set (Vick *et al*., 2018). Links between *mcrA* sequences and 16S rRNA gene OTUs were determined across metagenomic datasets, with plausible matches being selected based on the nearest BLAST type sequence matches for each except for mcra_30‐33, 44 and 45, which were identified by nearest BLAST nt database match due to lack of type sequence matches. 16S rRNA gene sequences were matched to the CSMB reference set using USEARCH v11.0.0667, at 97% identity (Supplementary Data Table S10; Supplementary Data Fig. S1).

### Water chemistry

For the Bowen 3, Surat 1 and Surat 2 samples used in this study, the water chemistry was analysed by Australia Laboratory Services, Environmental Division, Sydney, which is a NATA‐accredited (National Association of Testing Authorities, Australia; https://nata.com.au/) facility for environmental testing.

## Disclaimer

Any use of trade, firm, or product names is for descriptive purposes only and does not imply endorsement by the U.S. Government.

## Supporting information


**Fig. S1.** Proportion of archaeal 16S rRNA gene operational taxonomic units (OTUs) within each metagenomic dataset as detected with the Earth Microbiome Project primer sets (Apprill *et al*., 2015; Parada *et al*., 2016). Phylogenetic groupings of 16S rRNA OTUs by BLAST type sequence matching is provided down to the class level. Coal Seam Microbiome (CSMB; Vick *et al*., 2018) reference set matches found at >97% identity have been included.
**Fig. S2**. Two‐dimensional non‐metric multidimensional scaling (NMDS) plot of the archaeal 16S rRNA genes detected in the metagenomic datasets selected for this study. No archaeal 16S rRNA gene sequences were detected in the Powder River 10 dataset.

**Table S1.** *mcrA* sequences found using the four adjusted primer pairs (Table 1) trialled in the preliminary tests.
**Table S2**. Bathyarchaeota‐specific *mcrA* primer sets (Evans *et al*., 2015) trialled with Kelpie. Bases adjusted during the present study are underlined.
**Table S3**. Taxonomic details of each distinct mcrA gene by type sequence and by closest relatives found with NCBI BLAST. For further primer set details see Table 1 and Supplementary Data Table S12.
**Table S4**. Gene presence table displaying mcrA sequence counts produced from the MCR and Angel primer sets (Table 1). No mcrA sequences were detected in Powder River 50 and Power River 10. Grey boxes indicate sequences detected with the Angel primer set only; yellow boxes indicate sequences detected with the MCR primer set only. Numbers in brackets indicate the percent identity to the reference sequence (the reference sequences are available in FASTA format in the Supplementary Data).
**Table S5**. Coal seam formation water samples selected for use in this study.
**Table S6**. mcrA sequence diversity in the metagenomic datasets. Simpsons Index values shown are the highest detected by either primer set.*
**Table S7**. Archaeal 16S rRNA gene operational taxonomic unit (OTU) diversity in the metagenomic datasets.*
**Table S8**. Bulk water chemistry data for the Surat 1, Surat 2 and Bowen 3 coal seam formation water samples used in this study and Greenfield *et al*., 2019.
**Table S9**. Bulk water chemistry and dissolved gas data for the Nance, Flowers‐Goodale and Terret coal seam formation water subsurface environmental sampler samples used in this study, Barnhart *et al*., 2016, and Smith *et al*., 2021.
**Table S10**. Taxonomic details of each 16S rRNA gene operational taxonomic unit (OTU) by type sequence and by closest relatives found with BLAST. The Coal Seam Microbiome (CSMB; Vick *et al*., 2018) reference set match is included.
**Table S11**. Co‐occurrence of archaeal 16S rRNA gene OTUs from metagenomic datasets in the present study (marked *) and other basins from the Coal Seam Microbiome set (CSMB; Vick *et al*., 2018). Pale red = archaeon is present in data from CSMB set, dark red = archaeon is present in data from this study.
**Table S12**. mcrA primer sets trialled with Kelpie.

**Appendix S1**. List of the commands used with Kelpie and other programs.
*mcrA* MCR‐Angel primer consensus sequences:
A FASTA format file of final consensus *mcrA* sequences used in the present study, merged from the MCR and Angel primer set results. Sequence clustering was done at 99 %.
Archaeal 16S rRNA zero‐radius OTU sequences:
A FASTA format file of the archaeal 16S rRNA gene zero‐radius operational taxonomic unit sequences extracted from the thirteen metagenomes using Kelpie.
