# Supplementary material for: Methanogenic archaea in subsurface coal seams are biogeographically distinct: an analysis of metagenomically‐derived mcrA sequences
Source: Environ Microbiol. 2022 May 10;24(9):4065–78. doi: 10.1111/1462-2920.16014 (PMC9790511; doi:10.1111/1462-2920.16014)
Supplement: Supplementary file 1 — Fig. S1. Proportion of archaeal 16S rRNA gene operational taxonomic units (OTUs) within each metagenomic dataset as detected with the Earth Microbiome Project primer sets (Apprill et al., 2015; Parada et al., 2016). Phylogenetic groupings of 16S rRNA OTUs by BLAST type sequence matching is provided down to the class level. Coal Seam Microbiome (CSMB; Vick et al., 2018) reference set matches found at >97% identity have been included. Fig. S2. Two‐dimensional non‐metric multidimensional scaling (NMDS) plot of the archaeal 16S rRNA genes detected in the metagenomic datasets selected for this study. No archaeal 16S rRNA gene sequences were detected in the Powder River 10 dataset. [file EMI-24-4065-s002.zip › EMI_16014_Supplementary_data_computing_commands.docx]

Kelpie_v2 -f TWYGAYCARRTHTGGYT -r ACRTTCATNGCRTARTT Cel119_?.fastq Cel119_MCR.fa -mm 0
-length 520 -unfiltered -loose

Kelpie_v2 -f TWYGAYCARRTHTGGYT -r ACRTTCATNGCRTARTT Fav95_?.fastq Fav95_MCR.fa -mm 0
 -length 520 -unfiltered -loose

Kelpie_v2 -f TWYGAYCARRTHTGGYT -r ACRTTCATNGCRTARTT Tal69_?.fastq Tal69_MCR.fa -mm 0
-length 520 -unfiltered -loose

Kelpie_v2 -f TWYGAYCARRTHTGGYT -r ACRTTCATNGCRTARTT SRR12666050_?.fastq SRR12666050_MCR.fa -length 520 -mm 0 -unfiltered -loose

Kelpie_v2 -f TWYGAYCARRTHTGGYT -r ACRTTCATNGCRTARTT SRR11844785_?.fastq SRR11844785_MCR.fa -length 520 -mm 0 -unfiltered -loose

Kelpie_v2 -f TWYGAYCARRTHTGGYT -r ACRTTCATNGCRTARTT SRR11844784_?.fastq SRR11844784_MCR.fa -length 520 -mm 0 -unfiltered -loose

Kelpie_v2 -f TWYGAYCARRTHTGGYT -r ACRTTCATNGCRTARTT SRR11844737_?.fastq SRR11844737_MCR.fa -length 520 -mm 0 -unfiltered -loose

Kelpie_v2 -f TWYGAYCARRTHTGGYT -r ACRTTCATNGCRTARTT SRR11844740_?.fastq SRR11844740_MCR.fa -length 520 -mm 0 -unfiltered -loose

Kelpie_v2 -f TWYGAYCARRTHTGGYT -r ACRTTCATNGCRTARTT SRR13051708_?.fastq SRR13051708_MCR.fa -length 520 -mm 0 -unfiltered -loose

Kelpie_v2 -f TWYGAYCARRTHTGGYT -r ACRTTCATNGCRTARTT SRR13051709_?.fastq SRR13051709_MCR.fa -length 520 -mm 0 -unfiltered -loose

Kelpie_v2 -f TWYGAYCARRTHTGGYT -r ACRTTCATNGCRTARTT SRR13051710_?.fastq SRR13051710_MCR.fa -length 520 -mm 0 -unfiltered -loose

Kelpie_v2 -f TWYGAYCARRTHTGGYT -r ACRTTCATNGCRTARTT SRR2132206_?.fastq SRR2132206_MCR.fa -length 520 -mm 0 -unfiltered -loose

Kelpie_v2 -f TWYGAYCARRTHTGGYT -r ACRTTCATNGCRTARTT SRR3952189_?.fastq SRR3952189_MCR.fa -length 520 -mm 0 -unfiltered -loose

Kelpie_v2 -f GGYGGYGTMGGDTTCACMCARTA -r CRTTCATNGCRTARTTNGGRTAGT Cel119_?.fastq Cel119_Angel3.fa -length 480 -mm 2 -matches -unfiltered -strict

Kelpie_v2 -f GGYGGYGTMGGDTTCACMCARTA -r CRTTCATNGCRTARTTNGGRTAGT Fav95_?.fastq Fav95_Angel3.fa -length 480 -mm 2 -unfiltered -strict

Kelpie_v2 -f GGYGGYGTMGGDTTCACMCARTA -r CRTTCATNGCRTARTTNGGRTAGT Tal69_?.fastq Tal69_Angel3.fa -length 480 -mm 2 -unfiltered -strict

Kelpie_v2 -f GGYGGYGTMGGDTTCACMCARTA -r CRTTCATNGCRTARTTNGGRTAGT SRR12666050_?.fastq SRR12666050_Angel3.fa -length 480 -mm 2 -unfiltered -strict

Kelpie_v2 -f GGYGGYGTMGGDTTCACMCARTA -r CRTTCATNGCRTARTTNGGRTAGT SRR11844785_?.fastq SRR11844785_Angel3.fa -length 480 -mm 2 -unfiltered -strict

Kelpie_v2 -f GGYGGYGTMGGDTTCACMCARTA -r CRTTCATNGCRTARTTNGGRTAGT SRR11844784_?.fastq SRR11844784_Angel3.fa -length 480 -mm 2 -unfiltered -strict

Kelpie_v2 -f GGYGGYGTMGGDTTCACMCARTA -r CRTTCATNGCRTARTTNGGRTAGT SRR11844737_?.fastq SRR11844737_Angel3.fa -length 480 -mm 2 -unfiltered -strict

Kelpie_v2 -f GGYGGYGTMGGDTTCACMCARTA -r CRTTCATNGCRTARTTNGGRTAGT SRR11844740_?.fastq SRR11844740_Angel3.fa -length 480 -mm 2 -unfiltered -strict

Kelpie_v2 -f GGYGGYGTMGGDTTCACMCARTA -r CRTTCATNGCRTARTTNGGRTAGT SRR13051708_?.fastq SRR13051708_Angel3.fa -length 480 -mm 2 -unfiltered -strict

Kelpie_v2 -f GGYGGYGTMGGDTTCACMCARTA -r CRTTCATNGCRTARTTNGGRTAGT SRR13051709_?.fastq SRR13051709_Angel3.fa -length 480 -mm 2 -unfiltered -strict

Kelpie_v2 -f GGYGGYGTMGGDTTCACMCARTA -r CRTTCATNGCRTARTTNGGRTAGT SRR13051710_?.fastq SRR13051710_Angel3.fa -length 480 -mm 2 -unfiltered -strict

Kelpie_v2 -f GGYGGYGTMGGDTTCACMCARTA -r CRTTCATNGCRTARTTNGGRTAGT SRR2132206_?.fastq SRR2132206_Angel3.fa -length 480 -mm 2 -unfiltered -strict

Kelpie_v2 -f GGYGGYGTMGGDTTCACMCARTA -r CRTTCATNGCRTARTTNGGRTAGT SRR3952189_?.fastq SRR3952189_Angel3.fa -length 480 -mm 2 -unfiltered -strict

// cluster each of the sets of sequences returned by Kelpie at 99% to remove

// minor strain variation and small sequence error variants.

parallelforfiles *_Angel3.fa usearch11 -cluster_fast ?file -id 0.99 -centroids ?fname_c99.fa -sizeout -relabel ?fname_ -sort length

parallelforfiles *_MCR.fa usearch11 -cluster_fast ?file -id 0.99 -centroids
?fname_c99.fa -sizeout -relabel ?fname_ -sort length

// create single files for the clustered sequences from each primer

catseqs *_MCR_c99.fa MCR_c99_merged.fa

catseqs *_Angel3_c99.fa Angel3_c99_merged.fa

//merge all the mcrA sequences from both primers into a single file

catseqs *_MCR_c99.fa *_Angel3_c99.fa MCR_Angel3_c99_merged.fa

// and cluster at 99% to get a single set of consensus sequences for all mcrA genes

// found with either // primer and in any sample

usearch11 -cluster_fast MCR_Angel3_c99_merged.fa -id 0.99 -centroids MCR_Angel3_c99_consensus.fa -sizein -relabel MCR_Angel3_c99_ -sort length

// map the per-sample/primer sequences back to the overall consensus so we can see what

// genes occurred where

usearch11 -usearch_global .\MCR_c99_merged.fa -db .\MCR_Angel3_c99_consensus.fa -uc MCR_c99_consensus.uc -strand plus -id .99

usearch11 -usearch_global .\Angel3_c99_merged.fa -db .\MCR_Angel3_c99_consensus.fa -uc Angel3_c99_consensus.uc -strand plus -id .99

// turn the matches data into a convenient Gene Presence Table

GenerateGPTFromKelpieUC _MCR_ MCR_c99_consensus.uc MCR_c99_consensus_GPT.txt

GenerateGPTFromKelpieUC _Angel3_ Angel3_c99_consensus.uc Angel3_c99_consensus_GPT.txt
