# Supplementary material for: Methanogenic archaea in subsurface coal seams are biogeographically distinct: an analysis of metagenomically‐derived mcrA sequences
Source: Environ Microbiol. 2022 May 10;24(9):4065–78. doi: 10.1111/1462-2920.16014 (PMC9790511; doi:10.1111/1462-2920.16014)
Supplement: Supplementary file 2 — Table S1. mcrA sequences found using the four adjusted primer pairs (Table 1) trialled in the preliminary tests. Table S2. Bathyarchaeota‐specific mcrA primer sets (Evans et al., 2015) trialled with Kelpie. Bases adjusted during the present study are underlined. Table S3. Taxonomic details of each distinct mcrA gene by type sequence and by closest relatives found with NCBI BLAST. For further primer set details see Table 1 and Supplementary Data Table S12. Table S4. Gene presence table displaying mcrA sequence counts produced from the MCR and Angel primer sets (Table 1). No mcrA sequences were detected in Powder River 50 and Power River 10. Grey boxes indicate sequences detected with the Angel primer set only; yellow boxes indicate sequences detected with the MCR primer set only. Numbers in brackets indicate the percent identity to the reference sequence (the reference sequences are available in FASTA format in the Supplementary Data). Table S5. Coal seam formation water samples selected for use in this study. Table S6. mcrA sequence diversity in the metagenomic datasets. Simpsons Index values shown are the highest detected by either primer set.* Table S7. Archaeal 16S rRNA gene operational taxonomic unit (OTU) diversity in the metagenomic datasets.* Table S8. Bulk water chemistry data for the Surat 1, Surat 2 and Bowen 3 coal seam formation water samples used in this study and Greenfield et al., 2019. Table S9. Bulk water chemistry and dissolved gas data for the Nance, Flowers‐Goodale and Terret coal seam formation water subsurface environmental sampler samples used in this study, Barnhart et al., 2016, and Smith et al., 2021. Table S10. Taxonomic details of each 16S rRNA gene operational taxonomic unit (OTU) by type sequence and by closest relatives found with BLAST. The Coal Seam Microbiome (CSMB; Vick et al., 2018) reference set match is included. Table S11. Co‐occurrence of archaeal 16S rRNA gene OTUs from metagenomic datasets in the present study (marked *) and [file EMI-24-4065-s003.docx]

**Supplementary Data – Figures**


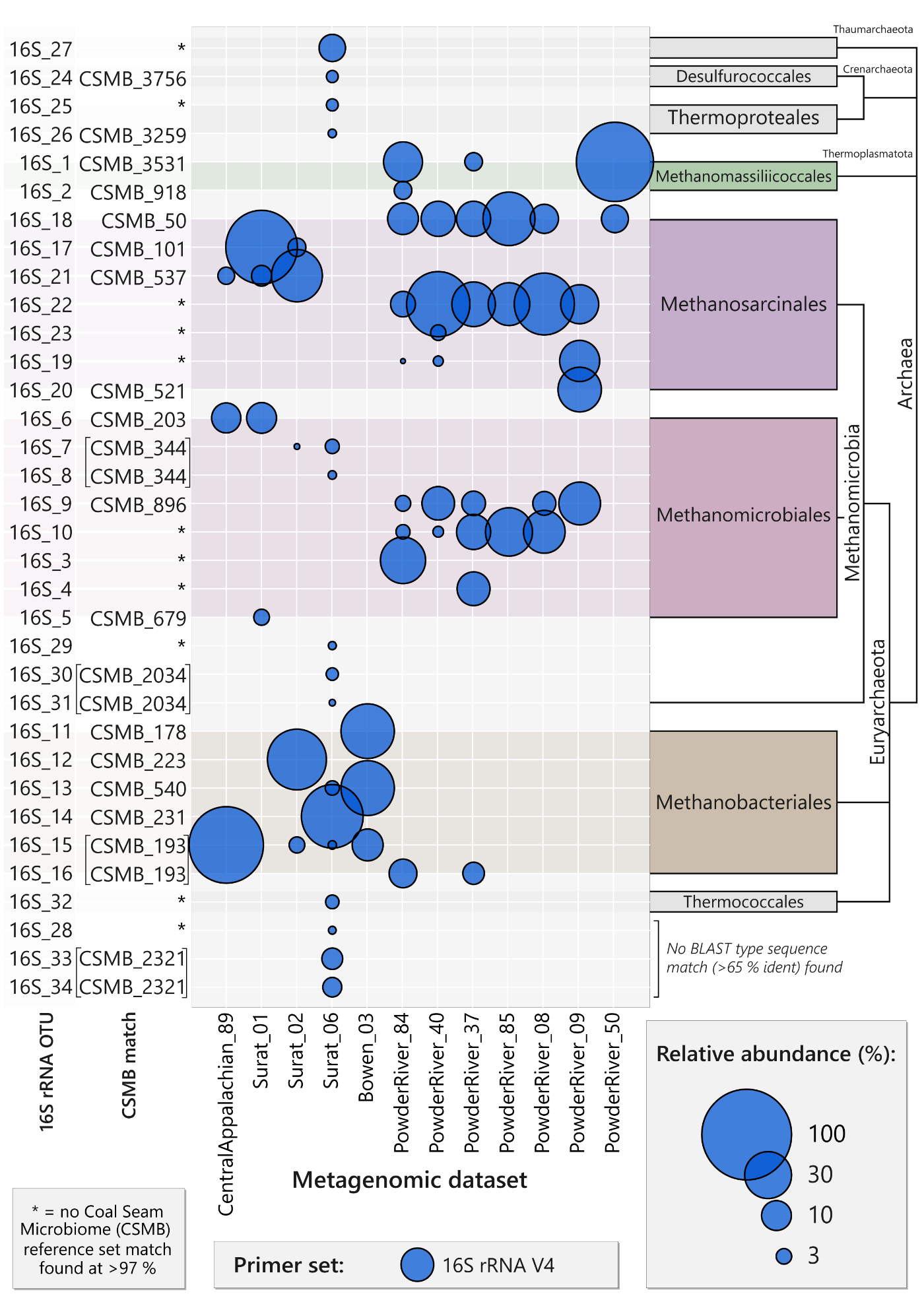


Figure S1: Proportion of archaeal 16S rRNA operational taxonomic units (OTUs) within each metagenomic dataset as detected with the Earth Microbiome Project primer sets (Apprill *et al.*, 2015; Parada *et al.*, 2016). Phylogenetic groupings of 16S rRNA OTUs by BLAST type sequence matching is provided down to the class level. Coal Seam Microbiome (CSMB; Vick et al. 2018) reference set matches found at >97 % identity have been included.


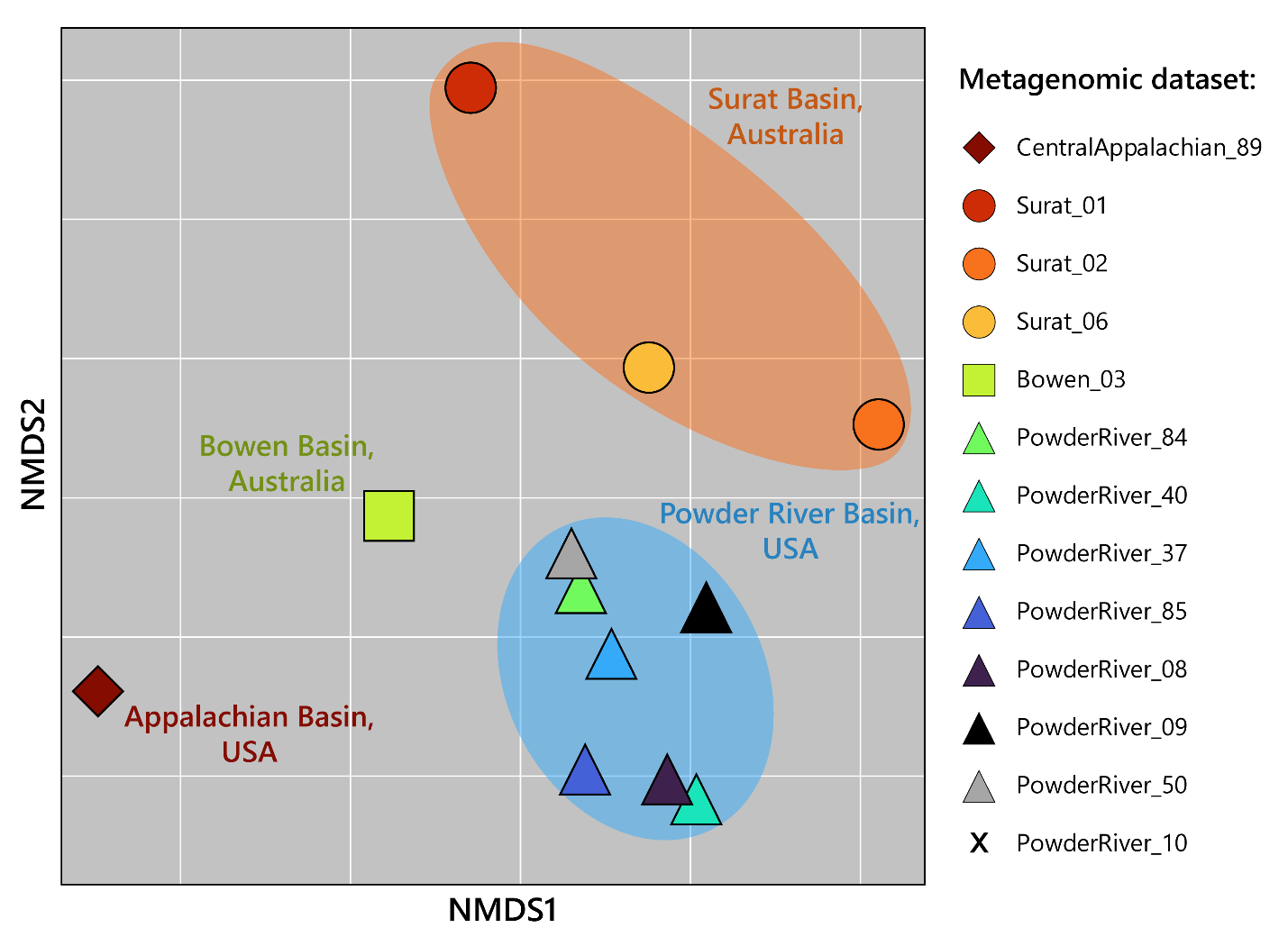


Figure S2: Two-dimensional non-metric multidimensional scaling (NMDS) plot of the archaeal 16S rRNA detected in the metagenomic datasets selected for this study. No archaeal 16S rRNA sequences were detected in the Powder River 10 dataset.
