# Supplementary material for: Methanogenic archaea in subsurface coal seams are biogeographically distinct: an analysis of metagenomically‐derived mcrA sequences
Source: Environ Microbiol. 2022 May 10;24(9):4065–78. doi: 10.1111/1462-2920.16014 (PMC9790511; doi:10.1111/1462-2920.16014)
Supplement: Supplementary file 3 — Appendix S1. List of the commands used with Kelpie and other programs. mcrA MCR‐Angel primer consensus sequences: A FASTA format file of final consensus mcrA sequences used in the present study, merged from the MCR and Angel primer set results. Sequence clustering was done at 99 %. Archaeal 16S rRNA zero‐radius OTU sequences: A FASTA format file of the archaeal 16S rRNA gene zero‐radius operational taxonomic unit sequences extracted from the thirteen metagenomes using Kelpie. [file EMI-24-4065-s001.docx]

**Supplementary Data – Tables**

| **Table S1: *mcrA* sequences found using the four adjusted primer pairs (Table 1) trialled in the preliminary tests.** | | | | | | | | | | | | |
| --- | --- | --- | --- | --- | --- | --- | --- | --- | --- | --- | --- | --- |
|  | **Surat 1** | | | | **Bowen 3** | | | | **Surat 2** | | | |
| **mcr#** | **ME** | **MCR** | **Angel** | **ML** | **ME** | **MCR** | **Angel** | **ML** | **ME** | **MCR** | **Angel** | **ML** |
| **1** |  |  |  |  | + | + | + | + |  |  |  |  |
| **2** |  |  |  |  |  | + | + | + | + | + | + | + |
| **3** |  |  |  |  | + | + | + |  |  |  |  |  |
| **4** |  |  |  |  |  |  |  |  |  | + | + | + |
| **5** |  |  |  |  |  |  |  |  | + | + | + | + |
| **6** |  |  |  |  | + | + |  |  | ++ | ++ |  |  |
| **7** |  |  |  |  | + | + | + | + |  |  |  |  |
| **8** |  |  |  |  |  |  |  |  | + | + | + | + |
| **9** |  | + | + | + |  |  |  |  |  | + | + | + |
| **10** | + | + | + | + |  |  |  |  | + | + | + | + |
| **11** |  | + | + | + |  |  |  |  |  |  |  |  |
| **12** |  |  |  |  |  |  |  |  |  | + | + |  |
| **13** | + | ++ | ++ | ++ |  |  |  |  |  |  |  |  |
| **14** | ++ | + | + | + |  |  |  |  |  |  |  |  |
| **15** |  | + |  |  |  |  |  |  |  |  |  |  |
| **16** | + | ++ | ++ | ++ |  |  |  |  |  |  |  |  |
|  | *+ = lower abundance; ++ = higher abundance; grey box = detected by MCR but not Angel* | | | | | | | | | | | |

| **Table S2: Bathyarchaeota-specific *mcrA* primer sets (Evans et al. 2015) trialled with Kelpie. Bases adjusted during the present study are underlined.** | | |
| --- | --- | --- |
| **Primer set** | **Modified sequence (5’ to 3’)** | **Original primer reference** |
| MCR-BA1-2 | forward-GACARYRYSWWWTGGYT | Springer et al. 1995 |
|  | reverse-GCSSTGAAWGATKSSWW |  |
| ME-BA1-2 | forward-GCYATGCAGAACACGATGGC | Hales et al. 1996 |
|  | reverse-TGAAWGATKSSWWYGGGKWGT |  |
| ML-BA1-2 | forward-GGCGGWATAGGRTTCWCRAAYACSRYWKCSGS | Luton et al. 2002 |
|  | reverse-STGAAWGATKSSWWYGGGKWGTT |  |

**Table S3: Taxonomic details of each distinct *mcrA* gene by type sequence and by closest relatives found with NCBI BLAST. For further primer set details see Table 1 and Supplementary Data Table S12. UASB = Up flow anaerobic sludge.**

| ***mcrA* sequence** | **BLAST assignment by type sequence match** | **BLAST closest relative** | | | |
| --- | --- | --- | --- | --- | --- |
|  |  | **Name** (accession) | **% cover** | **% ident** | **Environment** |
| mcra_1^a^ | Methanomassiliicoccaceae sp. | Uncultured archaeon  (LC002110) | 97 | 96.39 | Anaerobic granular sludge of UASB reactor treating sewage, Japan |
| mcra_2 | Methanosarcinaceae sp. | Uncultured archaeon  (KT314685) | 95 | 99.55 | Petroleum reservoir, China |
| mcra_3 | Methanocellales sp. | Uncultured archaeon  (LN716725) | 99 | 97.66 | Fen soil, Germany |
| mcra_4 | Methanocellales sp. | Uncultured archaeon  (JQ618193) | 100 | 94.93 | Rice rhizosphere soil, China |
| mcra_5 | Methanocellales sp. | Uncultured archaeon  (FJ754032) | 99 | 95.33 | Deep sea brine pool, Gulf of Mexico |
| mcra_6 | - | Uncultured archaeon  (GU085002) | 86 | 77.46 | Humic bog lake, Wisconsin, USA |
| mcra_7 | Methanobacteriaceae sp. | Uncultured archaeon  (FJ226712) | 99 | 74.95 | Biogas plant supplied with maize silage, barley grains |
| mcra_8 | *Methanosarcina* sp. | Uncultured archaeon  (KX233742) | 99 | 97.34 | Rice paddy soil, China |
| mcra_9 | *Methanolobus* sp. | *Methanolobus psychrophilus* (CP003083) | 100 | 95.51 | Zoige Wetland, Tibetan plateau |
| mcra_10^b^ | *Methanolobus* sp. | *Methanolobus psychrophilus* (CP003083) | 100 | 96.94 | Zoige Wetland, Tibetan plateau |
| mcra_11 | Methanomicrobiales sp. | Uncultured archaeon  (KF823442) | 100 | 95.97 | Rice paddy soil,  China |
| mcra_12 | Methanomicrobiales sp. | Uncultured archaeon  (LN716769) | 99 | 99.58 | Fen soil, Germany |
| mcra_13 | Methanomicrobiales sp. | Uncultured archaeon  (LN716673) | 99 | 97.46 | Fen soil, Germany |
| mcra_14^a^ | Methanomicrobiales sp. | Uncultured archaeon  (KR011381) | 100 | 98.82 | Lake sediment |
| mcra_15 | Methanomicrobiales sp. | Uncultured archaeon  (LN716673) | 99 | 98.51 | Fen soil, Germany |
| mcra_16 | Methanomicrobiales sp. | Uncultured archaeon  (KC184911) | 100 | 98.73 | Lake Pavin (90 m depth), France |
| mcra_17 | *Methanolinea* sp. | Uncultured archaeon  (KF595606) | 96 | 98.32 | Intertidal mudflat, Mai Po Nature Reserve, China |
| mcra_18 | *Methanolinea* sp. | Uncultured archaeon  (KF595606) | 96 | 98.53 | Intertidal mudflat, Mai Po Nature Reserve, China |
| mcra_19^a^ | *Methanolinea* sp. | Uncultured archaeon  (KF595606) | 100 | 97.09 | Intertidal mudflat, Mai Po Nature Reserve, China |
| mcra_20^a^ | *Methanolinea* sp. | Uncultured archaeon  (KF595606) | 100 | 96.41 | Intertidal mudflat, Mai Po Nature Reserve, China |
| mcra_21 | Methanomicrobiales sp. | Uncultured archaeon  (JN185013) | 96 | 96.84 | Soda lake sediments, Lonar Crater/Lake, India |
| mcra_22 | Methanomicrobiales sp. | Uncultured archaeon  (JN185013) | 96 | 96.84 | Soda lake sediments, Lonar Crater/Lake, India |
| mcra_23 | *Methanospirillum* sp. | Uncultured archaeon  (KX234795) | 96 | 93.29 | Coal seam gas well water, China |
| mcra_24 | Methanomicrobiales sp. | Uncultured archaeon  (JX648567) | 100 | 96.35 | Lei-Gong-Huo mud volcano, Taiwan |
| mcra_27 | Methanomicrobiaceae sp. | Uncultured archaeon  (JX097240) | 100 | 96.75 | Tidal flat sediments, German Wadden Sea |
| mcra_30^b^ | *Methanobacterium* sp.* | *Methanobacterium* sp.  (CP058560) | 100 | 98.28 | Water-sediment slurry, La Crouen spring, New Caledonia |
| mcra_31^b^ | *Methanobacterium* sp.* | *Methanobacterium* sp.  (CP058560) | 100 | 98.28 | Water-sediment slurry, La Crouen spring, New Caledonia |
| mcra_32^b^ | *Methanobacterium* sp.* | *Methanobacterium* sp.  (CP058560) | 100 | 97.21 | Water-sediment slurry, La Crouen spring, New Caledonia |
| mcra_33^b^ | *Methanobacterium* sp.* | *Methanobacterium* sp.  (CP058560) | 100 | 98.28 | Water-sediment slurry, La Crouen spring, New Caledonia |
| mcra_34 | *Methanobacterium* sp. | Uncultured archaeon  (KF761097) | 100 | 98.71 | Rice paddy soil, China |
| mcra_35 | *Methanothermobacter* sp. | *Methanothermobacter* sp. (CP043480) | 99 | 98.08 | Petroleum reservoir, China |
| mcra_36 | *Methanothermobacter* sp. | Uncultured bacterium  (JX942524) | 97 | 98.91 | Sediments, China |
| mcra_37 | *Methanobacterium* sp. | *Methanobacterium* sp. (CP022705) | 100 | 95.02 | Brine-fed electro-methanogenic bioreactor BR12, Japan |
| mcra_38 | *Methanobacterium* sp. | Uncultured archaeon  (EF117680) | 100 | 96.19 | Subsurface shale pore waters, USA |
| mcra_39 | *Methanobacterium* sp. | Uncultured archaeon  (MN392136) | 96 | 98.68 | Daqing oil reservoir, China |
| mcra_40 | *Methanobacterium* sp. | *Methanobacterium* sp.  (CP058560) | 100 | 99.79 | Water-sediment slurry, La Crouen spring, New Caledonia |
| mcra_41 | Methanobacteriales sp. | Uncultured archaeon  (JX853634) | 100 | 89.36 | Rice paddy soil, China |
| mcra_42 | Methanobacteriaceae sp. | Uncultured archaeon (KX234759) | 96 | 97.57 | Coal seam gas well water, China |
| mcra_43 | *Methanobacterium* sp. | Uncultured archaeon  (HE647307) | 99 | 96.79 | Earthworm gut, Brazil |
| mcra_44^b^ | Methanosarcinales sp.* | Uncultured archaeon (KJ595962) | 90 | 90.16 | High arsenic content aquifer, Inner Mongolia, China |
| mcra_45 | Methanosarcinales sp.* | Uncultured archaeon (KP342982) | 100 | 93.05 | Rice paddy soil, China |
| mcra_46 | Methanosarcinales sp. | Uncultured archaeon (KT314668) | 95 | 93.83 | Petroleum reservoir oil phase, China |
| mcra_47^a^ | Methanomicrobiales sp. | Uncultured archaeon (JX097257) | 100 | 95.96 | Tidal flat sediments, German Wadden Sea |
| mcra_48^b^ | Methanomicrobiales sp. | Uncultured archaeon (EF117668) | 100 | 97.36 | Subsurface shale pore water, USA |
| mcra_49^b^ | Methanomicrobiales sp. | Uncultured archaeon (JX430057) | 98 | 96.56 | Marsh soil, Min River estuary, China |

*Assignment is from nearest relatives in the BLAST nt database, not type sequence matches.

^a^Sequences detected with Angel primer set only.

^b^Sequences detected with MCR primer set only.

| **Table S4: Gene presence table displaying *mcrA* sequence counts produced from the MCR and Angel primer sets (Table 1). No *mcrA* sequences were detected in Powder River 50 and Power River 10. Grey boxes indicate sequences detected with the Angel primer set only; yellow boxes indicate sequences detected with the MCR primer set only. Numbers in brackets indicate the percent identity to the reference sequence (the reference sequences are available in fasta format in the Supplementary Data).** | | | | | | | | | | | | | | | | | | | | | | |
| --- | --- | --- | --- | --- | --- | --- | --- | --- | --- | --- | --- | --- | --- | --- | --- | --- | --- | --- | --- | --- | --- | --- |
|  | **MCR** | **Angel** | **MCR** | **Angel** | **MCR** | **Angel** | **MCR** | **Angel** | **MCR** | **Angel** | **MCR** | **Angel** | **MCR** | **Angel** | **MCR** | **Angel** | **MCR** | **Angel** | **MCR** | **Angel** | **MCR** | **Angel** |
|  | **Central Appalachian 89** | | **Surat 1** | | **Surat 2** | | **Surat 6** | | **Bowen 3** | | **Powder River 84** | | **Powder River 40** | | **Powder River 37** | | **Powder River 85** | | **Powder River 8** | | **Powder River 9** | |
| **mcra_1** |  |  |  |  |  |  |  |  |  |  |  | 22 (100) |  |  |  |  |  |  |  |  |  |  |
| **mcra_2** |  |  | 175 (100) | 171 (100) | 1704 (100) | 1819 (100) | 9 (100) |  |  |  |  |  |  |  |  |  |  |  |  |  |  |  |
| **mcra_3** |  |  |  |  |  |  |  |  |  |  | 16 (100) | 18 (100) | 171 (100) | 159 (100) | 14 (100) | 11 (100) | 20 (100) | 13 (100) | 9 (100) |  |  |  |
| **mcra_4** |  |  |  |  |  |  |  |  |  |  |  |  | 2 (100) | 2 (100) |  |  |  |  |  |  |  |  |
| **mcra_5** |  |  |  |  |  |  |  |  |  |  |  |  | 42 (100) | 36 (99.8) |  |  |  |  |  |  |  |  |
| **mcra_6** |  |  |  |  |  |  |  |  |  |  | 21 (100) |  |  | 8 (100) |  |  |  |  |  |  |  |  |
| **mcra_7** |  |  | 131 (100) | 162 (100) |  |  |  |  |  |  |  |  |  |  |  |  |  |  |  |  |  |  |
| **mcra_8** |  |  | 889 (100) | 858 (100) | 88 (100) | 86 (100) |  |  |  |  |  |  |  |  |  |  |  |  |  |  |  |  |
| **mcra_9** |  |  |  |  |  |  |  |  |  |  | 21 (100) | 16 (100) | 26 (100) | 27 (100) | 1 (100) |  | 10 (100) |  |  |  |  |  |
| **mcra_10** |  |  |  |  |  |  |  |  |  |  |  |  | 6 (100) |  |  |  | 4 (100) |  |  |  |  |  |
| **mcra_11** |  |  |  |  |  |  |  |  |  |  |  |  |  |  | 14 (99.4) | 7 (99.3) | 43 (99.4) | 38 (99.3) | 14 (100) | 8 (100) | 7 (99.4) | 7 (99.3) |
| **mcra_12** |  |  |  |  |  |  |  |  |  |  | 10 (100) | 14 (100) | 56 (99.8) | 45 (100) | 13 (100) | 13 (100) |  |  |  |  |  |  |
| **mcra_13** |  |  |  |  |  |  |  |  |  |  |  |  | 6 (100) | 2 (100) |  |  |  |  |  |  |  |  |
| **mcra_14** |  |  |  |  |  |  |  |  |  |  |  |  |  | 1 (100) |  |  |  |  |  |  |  |  |
| **mcra_15** |  |  |  |  |  |  |  |  |  |  |  |  | 2 (100) |  |  |  |  |  |  |  |  |  |
| **mcra_16** |  |  |  |  |  |  |  |  |  |  |  |  | 4 (100) | 1 (100) |  |  |  |  |  |  |  |  |
| **mcra_17** |  |  |  |  | 16 (100) | 14 (100) |  |  |  |  |  |  |  |  |  |  |  |  |  |  |  |  |
| **mcra_18** |  |  |  |  |  |  | 24 (100) | 65 (100) |  |  |  |  |  |  |  |  |  |  |  |  |  |  |
| **mcra_19** |  |  |  |  |  |  |  | 6 (100) |  |  |  |  |  |  |  |  |  |  |  |  |  |  |
| **mcra_20** |  |  |  |  |  |  |  | 3 (100) |  |  |  |  |  |  |  |  |  |  |  |  |  |  |
| **mcra_21** |  |  | 22 (100) | 32 (100) |  |  |  |  |  |  |  |  |  |  |  |  |  |  |  |  |  |  |
| **mcra_22** |  |  | 16 (100) | 7 (100) |  |  |  |  |  |  |  |  |  |  |  |  |  |  |  |  |  |  |
| **mcra_23** |  |  |  |  |  |  |  |  |  |  | 20 (100) | 19 (100) |  |  |  |  |  |  |  |  |  |  |
| **mcra_24** |  |  | 21 (100) | 26 (100) |  |  |  |  |  |  |  |  |  |  |  |  |  |  |  |  |  |  |
| **mcra_27** |  |  | 221 (100) | 212 (100) |  |  |  |  |  |  |  |  |  |  |  |  |  |  |  |  |  |  |
| **mcra_30** |  |  |  |  | 62 (99.6) |  | 119 (100) |  | 39 (99.6) |  |  |  |  |  |  |  |  |  |  |  |  |  |
| **mcra_31** |  |  |  |  | 13 (99.4) |  | 3 (100) |  |  |  |  |  |  |  |  |  |  |  |  |  |  |  |
| **mcra_32** | 13 (100) |  |  |  |  |  |  |  |  |  |  |  |  |  |  |  |  |  |  |  |  |  |
| **mcra_33** | 11 (100) |  |  |  |  |  |  |  |  |  |  |  |  |  |  |  |  |  |  |  |  |  |
| **mcra_34** |  |  |  |  | 1029 (100) | 1104 (100) |  |  |  |  |  |  |  |  |  |  |  |  |  |  |  |  |
| **mcra_35** |  |  |  |  |  |  |  |  | 125 (100) | 111 (100) |  |  |  |  |  |  |  |  |  |  |  |  |
| **mcra_36** |  |  |  |  |  |  |  |  | 102 (100) | 114 (100) |  |  |  |  |  |  |  |  |  |  |  |  |
| **mcra_37** |  |  |  |  |  |  | 35 (100) | 34 (100) |  |  |  |  |  |  |  |  |  |  |  |  |  |  |
| **mcra_38** |  |  |  |  |  |  |  |  |  |  | 12 (100) | 11 (100) |  |  |  |  |  |  |  |  |  |  |
| **mcra_39** |  |  |  |  |  |  | 493 (100) | 590 (100) |  |  |  |  |  |  |  |  |  |  |  |  |  |  |
| **mcra_40** | 39 (100) | 51 (100) |  |  | 77 (100) | 72 (100) | 154 (99.4) | 182 (100) | 72 (100) | 64 (100) |  |  |  |  |  |  |  |  |  |  |  |  |
| **mcra_41** |  |  |  |  |  |  | 30 (99.8) | 36 (99.8) | 261 (100) | 261 (100) |  |  |  |  |  |  |  |  |  |  |  |  |
| **mcra_42** |  |  |  |  | 980 (100) | 938 (100) |  |  |  |  |  |  |  |  |  |  |  |  |  |  |  |  |
| **mcra_43** |  |  |  |  | 29 (100) | 57 (100) |  |  |  |  |  |  |  |  |  |  |  |  |  |  |  |  |
| **mcra_44** |  |  |  |  |  |  |  |  |  |  |  |  |  |  |  |  |  |  |  |  | 6 (100) |  |
| **mcra_45** |  |  |  |  |  |  |  |  |  |  |  |  | 14 (100) | 12 (100) |  |  |  |  |  |  | 22 (100) |  |
| **mcra_46** | 2 (100) |  |  |  |  |  |  |  |  |  |  |  |  |  |  |  |  |  |  |  |  |  |
| **mcra_47** |  |  |  | 1 (100) |  |  |  |  |  |  |  |  |  |  |  |  |  |  |  |  |  |  |
| **mcra_48** | 5 (100) |  |  |  |  |  |  |  |  |  |  |  |  |  |  |  |  |  |  |  |  |  |
| **mcra_49** |  |  |  |  |  |  |  |  |  |  |  |  |  |  |  |  |  |  | 3 (100) |  |  |  |

| **Table S5: Coal seam formation water samples selected for use in this study**. | | | |  |
| --- | --- | --- | --- | --- |
| **Dataset name used in this study** | **NCBI sequence run**^1^  **IMG/M Genome ID**^2^ | **Geological basin, country** | **Formation** | **Submission author & year** |
| CentralAppalachian_89 | SRR3952189^1^ | Central  Appalachian  Basin, USA | Pocahontas No. 3 coal seam | D. Ross, 2016 |
| Surat_01 | *Not applicable*^3^ | Surat Basin, Australia | Walloon Subgroup | Greenfield *et al*. 2019 & this study |
| Surat_02 | *Not applicable*^3^ |  |  |  |
| Surat_06 | SRR2132206^1^ |  |  | D. Parks, 2015 |
| Bowen_03 | *Not applicable*^3^ | Bowen Basin, Australia | Bandanna Formation | Greenfield *et al*. 2019 & this study |
| PowderRiver_10 | SRR13051710^1^ | Powder River Basin, USA | Nance coal seam | M.W. Fields, 2020 |
| PowderRiver_09 | SRR13051709^1^ |  | Flowers-Goodale coal seam |  |
| PowderRiver_50^a^ | SRR12666050^1^  3300037286^2^ |  |  | E. Barnhart, 2019 |
| PowderRiver_40^b^ | SRR11844740^1^  3300037047^2^ |  |  |  |
| PowderRiver_37^c^ | SRR11844737^1^  3300037046^2^ |  |  |  |
| PowderRiver_85^c^ | SRR11844785^1^  3300037048^2^ |  |  |  |
| PowderRiver_84 | SRR11844784^1^  3300036987^2^ |  | Terret coal seam |  |
| PowderRiver_08 | SRR13051708^1^ |  |  | M.W. Fields, 2020 |

^1^Available from the National Center for Biotechnology Information (NCBI) Genbank database (<https://www.ncbi.nlm.nih.gov/genbank/>)

^2^Available from the Integrated Microbial Genomes and Microbiomes (IMG/M) database (<https://jgi.doe.gov/>)

^3^Available from the Commonwealth Scientific and Industrial Research Organisation (CSIRO) Data Access Portal (<https://data.csiro.au/collection/csiro:33617v1>). ‘W1’ = Surat_01; ‘W2’ = Bowen_03; ‘W3’ = Surat_02.

^a^From well FGM in the Flowers-Goodale coal seam

^b^From well FGP in the Flowers-Goodale coal seam

^c^From well FG11 in the Flowers-Goodale coal seam

**Table S6: *mcrA* sequence diversity in the metagenomic datasets. Simpsons Index values shown are the highest detected by either primer set.***

| **Metagenomic dataset** | **Richness** | **Methanogen biodiversity**  (Simpsons Index 1-D) | **Major orders**  (>30 % of reads) |
| --- | --- | --- | --- |
| Central Appalachian 89 | 5 | 0.62 | Methanobacteriales |
| Surat 1 | 8 | 0.61 | Methanosarcinales |
| Surat 2 | 9 | 0.69 | Methanobacteriales, Methanosarcinales |
| Surat 6 | 10 | 0.62 | Methanobacteriales |
| Bowen 3 | 5 | 0.71 | Methanobacteriales |
| Powder River 84 | 7 | 0.83 | Methanomicrobiales |
| Powder River 40 | 12 | 0.67 | Methanocellales |
| Powder River 37 | 4 | 0.68 | Methanomicrobiales, Methanocellales |
| Powder River 85 | 4 | 0.60 | Methanomicrobiales, Methanocellales^b^ |
| Powder River 8 | 3 | 0.58 | Methanomicrobiales, Methanocellales^b^ |
| Powder River 9 | 3 | 0.53 | Methanomicrobiales^a^, Methanosarcinales^b^ |

*No *mcrA* sequences were detected in the Powder River 10 or Powder River 50 datasets.

^a^Dominant in Angel primer set-generated reads only.

^b^Dominant in MCR primer set-generated reads only.

**Table S7: Archaeal 16S rRNA gene operational taxonomic unit (OTU) diversity in the metagenomic datasets.***

| **Metagenomic dataset** | **OTU richness** | **Archaeal Biodiversity** (Simpsons Index 1-D) | **Major orders**  (>30 % of reads) |
| --- | --- | --- | --- |
| Central Appalachian 89 | 3 | 0.30 | Methanobacteriales |
| Surat 1 | 4 | 0.39 | Methanosarcinales |
| Surat 2 | 5 | 0.57 | Methanosarcinales, Methanobacteriales |
| Surat 6 | 16 | 0.65 | Methanobacteriales |
| Bowen 3 | 3 | 0.61 | Methanobacteriales |
| Powder River 84 | 9 | 0.81 | Methanomicrobiales |
| Powder River 40 | 6 | 0.58 | Methanosarcinales |
| Powder River 37 | 7 | 0.82 | Methanosarcinales, Methanomicrobiales |
| Powder River 85 | 3 | 0.66 | Methanosarcinales, Methanomicrobiales |
| Powder River 8 | 4 | 0.63 | Methanosarcinales, Methanomicrobiales |
| Powder River 9 | 4 | 0.76 | Methanosarcinales |
| Powder River 50 | 2 | 0.20 | Methanomassiliicoccales |

*No archaeal 16S rRNA gene sequences were detected in the Powder River 10 dataset.

| **Table S8: Bulk water chemistry data for the Surat 1, Surat 2 and Bowen 3 coal seam formation water samples used in this study and Greenfield et al. 2019.** | | | | | |
| --- | --- | --- | --- | --- | --- |
| **Basin** | | Surat | Surat | Surat | Bowen |
| **Sample name** | | **Surat_01** | **Surat_02** | **Surat_02** | **Bowen_03** |
| **Sampling date** | | 2/06/2015 | 7/05/2015 | 25/09/2015 | 2/10/2015 |
| Comments | | Good | Good | Good | Good |
| pH |  | 8.83 | 8.4 | 8.39 | 8.78 |
| Electrical Conductivity @ 25°C | µS/cm | 9410 | 3980 | 4210 | 1960 |
| **Alkalinity by PC Titrator** | | | | | |
| Hydroxide Alkalinity as CaCO_3_ | mg/L | <1 | <1 | <1 | <1 |
| Carbonate Alkalinity as CaCO_3_ | mg/L | 237 | 80 | 40 | 102 |
| Bicarbonate Alkalinity as CaCO_3_ | mg/L | 1140 | 1550 | 1780 | 759 |
| Total Alkalinity as CaCO_3_ | mg/L | 1380 | 1630 | 1820 | 860 |
| **Dissolved Major Anions** | | | | | |
| Silicon as SiO_2_ | mg/L | 17.3 | 26.5 | 28.1 | nd |
| **Total Major Anions** | | | | | |
| Sulfur as S | mg/L | <1 | <1 | <1 | <1 |
| **Sulfate (Turbidimetric) as SO_4_^2-^ by DA** | | | | | |
| Sulfate as SO_4_ - Turbidimetric | mg/L | <25 | <25 | 1 | <1 |
| **Chloride by Discrete Analyser** | | | | | |
| Chloride | mg/L | 1990 | 514 | 544 | 165 |
| **Dissolved Major Cations** | | | | | |
| Calcium | mg/L | nd | nd | nd | 3 |
| Magnesium | mg/L | 5 | <1 | <1 | <1 |
| Sodium | mg/L | 1940 | 1010 | 1000 | 503 |
| Potassium | mg/L | 10 | 4 | 4 | 2 |
| **Dissolved Metals by ICP-MS** | | | | | |
| Iron | mg/L | 1.03 | 0.12 | 0.22 | 0.17 |
| **Total Metals by ICP-MS** | | | | | |
| Barium | mg/L | 1.92 | 0.621 | 0.614 | 0.138 |
| Lithium | mg/L | 0.119 | 0.056 | 0.068 | 0.185 |
| Strontium | mg/L | 2.88 | 0.833 | 0.78 | 0.212 |
| **Ferrous Iron by Discrete Analyser** | | | | | |
| Ferrous Iron | mg/L | 3.09 | <0.05 | <0.05 | 0.06 |
| **Fluoride by PC Titrator** | | | | | |
| Fluoride | mg/L | 1.8 | 5.2 | 4.5 | nd |
| **Nitrite as N by Discrete Analyser** | | | | | |
| Nitrite as N | mg/L | <0.01 | <0.01 | <0.01 | nd |
| **Nitrate as N by Discrete Analyser** | | | | | |
| Nitrate as N | mg/L | 0.78 | <0.01 | 0.05 | nd |
| **Nitrite plus Nitrate as N (NO*_x_*) by Discrete Analyser** | | | | | |
| Nitrite + Nitrate as N | mg/L | 0.78 | <0.01 | 0.05 | 0.03 |
| **Total Kjeldahl Nitrogen By Discrete Analyser** | | | | | |
| Total Kjeldahl Nitrogen as N | mg/L | 1.4 | 1 | 1 | 0.5 |
| **Total Nitrogen as N (TKN + NO*_x_*) by Discrete Analyser** | | | | | |
| Total Nitrogen as N | mg/L | 2.2 | 1 | 1 | 0.5 |
| **Total Phosphorus as P by Discrete Analyser** | | | | | |
| Total Phosphorus as P | mg/L | 0.02 | 0.04 | 0.04 | 0.04 |
| **Reactive Phosphorus as P by Discrete Analyser** | | | | | |
| Reactive Phosphorus as P | mg/L | 0.02 | 0.04 | 0.02 | 0.02 |
| **Ionic Balance** | | | | | |
| Total Anions | mg/L | nd | nd | nd | 21.8 |
| Total Cations | mg/L | nd | nd | nd | 22.1 |
| Ionic Balance | % | nd | nd | nd | 0.49 |

nd = no data

| **Table S9: Bulk water chemistry and dissolved gas data for the Nance, Flowers-Goodale and Terret coal seam formation water subsurface environmental sampler samples used in this study, Barnhart et al. 2016 and Smith et al. 2021.** | | | | | | | |
| --- | --- | --- | --- | --- | --- | --- | --- |
| **Basin** | | Powder River | Powder River | Powder River | Powder River | Powder River | Powder River |
| **Coal seam** | | **Nance** | **Flowers-Goodale** | **Flowers-Goodale** | **Flowers-Goodale** | **Flowers-Goodale** | **Terret** |
| **Well name** | | **N11** | **FG09** | **FGM** | **FGP** | **FG11** | **T11** |
| **Correlating sample name** | | **Powder River_10** | **Powder River_09** | **Powder River_50** | **Powder River_40** | **Powder River_37 and 85** | **Powder River_84 and 08** |
| **Sampling date** | | 7/26/2014 | 10/5/2011 | 6/11/2019 | 6/5/2018 | 7/23/2014 | 11/15/2011 |
| pH | | 8.0 | 8.1 | nd | nd | 8.1 | 8.3 |
| Temp | °C | 16.8 | 15.9 | nd | nd | 18.2 | nd |
| Sulfate | mM | 22.5 | 0.09 | 9.44 | 0.06 | 0.05 | 0.09 |
| Alkalinity | meq/kg | 14.49 | 22.96 | nd | 23.5 | 23.33 | 17.81 |
| **Dissolved gasses** |  |  |  |  |  |  |  |
| CO_2_ | mol % | 2.69 | 0.75 | 4.33 | 0.88 | 1.12 | 0.48 |
| C_1_ (methane) | mol % | 0.94 | 86.37 | 73.91 | 90.2 | 88.45 | 76.41 |
| C_2_ or higher chain hydrocarbons | mol % | nd | 0.008 | 0.0078 | 0.0096 | 0.013 | 0.004 |

nd = no data

| **Table S10: Taxonomic details of each 16S rRNA gene operational taxonomic unit (OTU) by type sequence and by closest relatives found with BLAST. The Coal Seam Microbiome (CSMB; Vick et al. 2018) reference set match is included.** | | | | | | |
| --- | --- | --- | --- | --- | --- | --- |
| **16S OTU**  CSMB match | **BLAST assignment by type sequence match** | **BLAST closest relatives** | | | | |
|  |  | **Name** | **% cover** | **% ident** | **Accession** | **Environment** |
| **16S_1**  CSMB_3531 | **Methanomassiliicoccaceae sp.** | Uncultured Thermoplasmatales sp. | 100 | 100 | LN796264 | Groundwater, Netherlands |
| **16S_2**  CSMB_918 | ***Methanomassiliicoccus* sp.** | Uncultured Methanomassiliicoccales sp. | 100 | 100 | MW386094 | Anaerobic digester |
| **16S_3**  * | ***Methanospirillum* sp.** | Uncultured *Methanospirillum* sp. | 100 | 100 | LT844368 | Oil-field, China |
| **16S_4**  * | ***Methanospirillum* sp.** | Uncultured *Methanospirillum* sp. | 100 | 99.21 | LT844368 | Oil-field, China |
| **16S_5**  CSMB_679 | ***Methanospirillum hungatei*** | Uncultured *Methanospirillum* sp. | 100 | 100 | EU812213 | Coal seam microbial community, Australia |
| **16S_6**  CSMB_203 | ***Methanocalculus pumilus*** | Uncultured *Methanocalculus* sp. | 100 | 100 | MN435002 | Petroleum reservoir production water, Karamay, Xinjiang, China |
| **16S_7**  CSMB_344 | ***Methanolinea* sp.** | *Methanolinea* sp. | 100 | 99.21 | JN836394 | Deep groundwater, Japan |
| **16S_8**  CSMB_344 | ***Methanolinea* sp.** | Uncultured archaeon | 100 | 100 | AB924389 | Deep groundwater, Japan |
| **16S_9**  CSMB_896 | ***Methanoregula formicica*** | Uncultured Methanomicrobiales sp. | 100 | 100 | LN796343 | Groundwater, Netherlands |
| **16S_10**  * | ***Methanoregula* sp.** | Uncultured Methanomicrobiales sp. | 100 | 100 | LN796202 | Groundwater, Netherlands |
| **16S_11**  CSMB_178 | ***Methanothermobacter thermautotrophicus*** | *Methanothermobacter thermautotrophicus* | 100 | 100 | MT178044 | Coal bed methane reservoir, India |
| **16S_12**  CSMB_223 | ***Methanobacterium subterraneum*** | *Methanobacterium subterraneum* | 100 | 100 | CP017768 | Deep subseafloor coal beds, offshore Japan |
| **16S_13**  CSMB_540 | ***Methanobacterium aarhusense*** | Uncultured *Methanobacterium* sp. | 100 | 100 | KR017724 | Oilfield production water, China |
| **16S_14**  CSMB_231 | ***Methanobacterium alcaliphilum*** | Uncultured Methanobacteriaceae sp. | 100 | 100 | MG001771 | Coal bed methane reservoir, India |
| **16S_15**  CSMB_193 | ***Methanobacterium alcaliphilum*** | *Methanobacterium* sp. | 100 | 100 | LC183834 | Subseafloor hydrate-bearing sediments, Nankai Trough, Japan |
| **16S_16**  CSMB_193 | ***Methanobacterium flexile*** | Uncultured *Methanobacterium* sp. | 100 | 100 | KY386106 | Abattoir water, Nigeria |
| **16S_17**  CSMB_101 | ***Methanosarcina barkeri*** | *Methanosarcina mazei* | 100 | 100 | CP042908 | Zoige wetland, Tibetan Plateau |
| **16S_18**  CSMB_50 | ***Methanolobus psychrophilus*** | Uncultured *Methanolobus* sp. | 100 | 100 | MH202882 | Petroleum reservoir production water, Karamay, Xinjiang, China |
| **16S_19**  * | **Methermicoccaceae sp.** | Uncultured archaeon | 100 | 100 | AB671634 | 140 m depth borehole, Japan |
| **16S_20**  CSMB_521 | **Methermicoccaceae sp.** | Uncultured archaeon | 100 | 100 | MK076113 | Lake sediment, China |
| **16S_21**  CSMB_537 | ***Methanosaeta* sp.** | Uncultured *Methanosaeta* sp. | 100 | 100 | MT445076 | Coal seam gas reservoir, Surat Basin, Australia |
| **16S_22**  * | ***Methanothrix soehngenii*** | Uncultured *Methanosaeta* sp. | 100 | 100 | MK246962 | Activated sludge metagenome |
| **16S_23**  * | ***Methanothrix soehngenii*** | Uncultured Methanosarcinales sp. | 100 | 100 | LN796409 | Groundwater, Netherlands |
| **16S_24**  CSMB_3756 | **Desulfurococcaceae sp.** | Uncultured Thermoproteales sp. | 96 | 100 | MF897010 | Coal seam environment, Australia |
| **16S_25**  * | **Thermofilaceae sp.** | Uncultured archaeon | 98 | 98.4 | KC925929 | Lake Kivu Sediment, core KS20, 450 m depth, Africa |
| **16S_26**  CSMB_3259 | **Thermofilaceae sp.** | Uncultured archaeon | 98 | 98.8 | KC925929 | Tropical marine sediment, around 20m water depth, India |
| **16S_27**  * | ***Conexivisphaeria* sp.** | Uncultured archaeon | 100 | 90.16 | AB369210 | Riser drilling mud fluid, offshore Japan |
| **16S_28**  * | **Uncultured archaeon** | Uncultured archaeon | 100 | 90.16 | JQ817013 | Subseafloor sediment at the Good Weather Ridge, Taiwan |
| **16S_29**  * | **Methanomicrobia sp.** | Uncultured archaeon | 100 | 95.67 | KJ655098 | Pinus pinaster tree, Portugal |
| **16S_30**  CSMB_2034 | **Methanomicrobia sp.** | Uncultured archaeon | 100 | 98.03 | AB679514 | Yunohama hot spring, Japan |
| **16S_31**  CSMB_2034 | **Methanomicrobia sp.** | Uncultured archaeon | 100 | 97.64 | AB679514 | Yunohama hot spring, Japan |
| **16S_32**  * | **Thermococcales sp.** | Uncultured archaeon | 99 | 96.05 | AB622747 | High arsenic aquifer, Inner Mongolia, China |
| **16S_33**  CSMB_2321 | **Uncultured archaeon** | Uncultured archaeon | 100 | 96.46 | AB094545 | Subseafloor sediments from Sea of Okhotsk, Japan |
| **16S_34**  CSMB_2321 | **Uncultured archaeon** | Uncultured archaeon | 100 | 96.46 | JQ816398 | Subseafloor sediment at the Fangliao Canyon, Taiwan |

*No CSMB reference set match found at >97 % identity.

| **Table S11: Co-occurrence of archaeal 16S rRNA gene operational taxonomic units (OTUs) from metagenomic datasets in the present study (marked *) and other basins from the Coal Seam Microbiome set (CSMB; Vick et al. 2018).**  Pale red = archaeon is present in data from CSMB set, dark red = archaeon is present in data from this study. | | | | | | | | | | | | | | | | | | | | |
| --- | --- | --- | --- | --- | --- | --- | --- | --- | --- | --- | --- | --- | --- | --- | --- | --- | --- | --- | --- | --- |
|  | | Australia | | | | | | | | India | China | | | Japan | United States of America | | | | | Canada |
| CSMB reference | BLAST assignment in CSMB set | Sydney Basin | Sydney Basin | Sydney Basin | Surat Basin | Surat Basin | Surat Basin* | Bowen Basin | Bowen Basin* | Damodar Basin | Jingmen-Dangyang Basin | Ordos Basin | Ordos Basin | Ishikari Basin | Cherokee Basin | Illinois Basin | Powder River Basin | Powder River Basin* | Central Appalachian Basin* | Western Canadian Sedimentary Basin |
| CSMB_3259 | Uncultured Desulfurococcales (MF896559) |  |  |  |  |  |  |  |  |  |  |  |  |  |  |  |  |  |  |  |
| CSMB_3756 | Uncultured Thermoproteales (MF897010) |  |  |  |  |  |  |  |  |  |  |  |  |  |  |  |  |  |  |  |
| CSMB_2321 | Uncultured Thermoprotei (ACSM_1880) |  |  |  |  |  |  |  |  |  |  |  |  |  |  |  |  |  |  |  |
| CSMB_193 | Uncultured Methanobacterium (AB294252.1) |  |  |  |  |  |  |  |  |  |  |  |  |  |  |  |  |  |  |  |
| CSMB_223 | Uncultured Methanobacterium (KT596091.1) |  |  |  |  |  |  |  |  |  |  |  |  |  |  |  |  |  |  |  |
| CSMB_231 | Uncultured Methanobacterium (KT596099.1) |  |  |  |  |  |  |  |  |  |  |  |  |  |  |  |  |  |  |  |
| CSMB_540 | Uncultured Methanobacterium (MF894001) |  |  |  |  |  |  |  |  |  |  |  |  |  |  |  |  |  |  |  |
| CSMB_178 | Uncultured Methanothermobacter (EU073827.2) |  |  |  |  |  |  |  |  |  |  |  |  |  |  |  |  |  |  |  |
| CSMB_203 | Uncultured Methanocalculus (KT596070.1) |  |  |  |  |  |  |  |  |  |  |  |  |  |  |  |  |  |  |  |
| CSMB_344 | Uncultured Methanolinea (KT596221.1) |  |  |  |  |  |  |  |  |  |  |  |  |  |  |  |  |  |  |  |
| CSMB_896 | Uncultured Methanoregula (MF894343) |  |  |  |  |  |  |  |  |  |  |  |  |  |  |  |  |  |  |  |
| CSMB_679 | Uncultured Methanospirillum (MF894133) |  |  |  |  |  |  |  |  |  |  |  |  |  |  |  |  |  |  |  |
| CSMB_50 | Uncultured Methanolobus (AB294253.1) |  |  |  |  |  |  |  |  |  |  |  |  |  |  |  |  |  |  |  |
| CSMB_101 | Uncultured Methanosarcina (AB294254.1) |  |  |  |  |  |  |  |  |  |  |  |  |  |  |  |  |  |  |  |
| CSMB_537 | Uncultured Methanothrix (MF893998) |  |  |  |  |  |  |  |  |  |  |  |  |  |  |  |  |  |  |  |
| CSMB_521 | Uncultured Methanosarcinales (Kirk_157) |  |  |  |  |  |  |  |  |  |  |  |  |  |  |  |  |  |  |  |
| CSMB_918 | Uncultured Methanomassiliicoccus (MF894363) |  |  |  |  |  |  |  |  |  |  |  |  |  |  |  |  |  |  |  |
| CSMB_3531 | Uncultured Methanomassiliicoccus (MF896808) |  |  |  |  |  |  |  |  |  |  |  |  |  |  |  |  |  |  |  |
| CSMB_2034 | Uncultured Archaea (ACSM_1694) |  |  |  |  |  |  |  |  |  |  |  |  |  |  |  |  |  |  |  |
|  | References: | Beckmann et al. 2016 | Li et al. 2008 | Vick et al. 2018 | Li et al. 2008 | Vick et al. 2018 | *This study | Vick et al. 2018 | *This study | Singh et al. 2011 | Wei et al. 2013 & 2014 | Guo et al. 2012 | Tang et al. 2012 | Shimizu et al. 2007 | Kirk et al. 2015 | Strapoc et al. 2008 | Green et al. 2008 | *This study | *This study | Penner et al. 2010 |

| **Table S12: *mcrA* primer sets trialled with Kelpie.** | | |
| --- | --- | --- |
| **Primer set** | **Sequence (5’ to 3’)** | **Reference** |
| MCR | forward-TAYGAYCARATHTGGYT | Springer et al. 1995 |
|  | reverse-ACRTTCATNGCRTARTT |  |
| ME | forward-GCMATGCARATHGGWATGTC | Hales et al. 1996 |
|  | reverse-TCATKGCRTAGTTDGGRTAGT |  |
| ML | forward-GGTGGTGTMGGATTCACACARTAYGCWACAGC | Luton et al. 2002 |
|  | reverse-TTCATTGCRTAGTTWGGRTAGTT |  |
| mlas-mod – F  *mcrA*-rev – R* | forward-GGYGGTGTMGGDTTCACMCARTA | Angel et al. 2012 |
|  | reverse-CGTTCATBGCGTAGTTVGGRTAGT |  |

*Referred to as the ‘Angel’ primer set in the present study, after the lead author of the 2012 study.
